# Ti(III) Catalysts for CO_2_/Epoxide Copolymerization at Unusual Ambient Pressure Conditions

**DOI:** 10.1021/acs.inorgchem.3c01249

**Published:** 2023-08-31

**Authors:** Ignacio Sancho, Marta Navarro, Marc Montilla, Pedro Salvador, Cristina Santamaría, Josep M. Luis, Alberto Hernán-Gómez

**Affiliations:** † Departamento de Química Orgánica y Química Inorgánica, Instituto de Investigación Química “Andrés M. del Río” (IQAR), Universidad de Alcalá, Campus Universitario, E-28805 Alcalá de Henares, Madrid, Spain; ‡ Institute of Computational Chemistry and Catalysis and Department of Chemistry, University of Girona, Campus de Montilivi, 17003 Girona, Catalonia, Spain

## Abstract

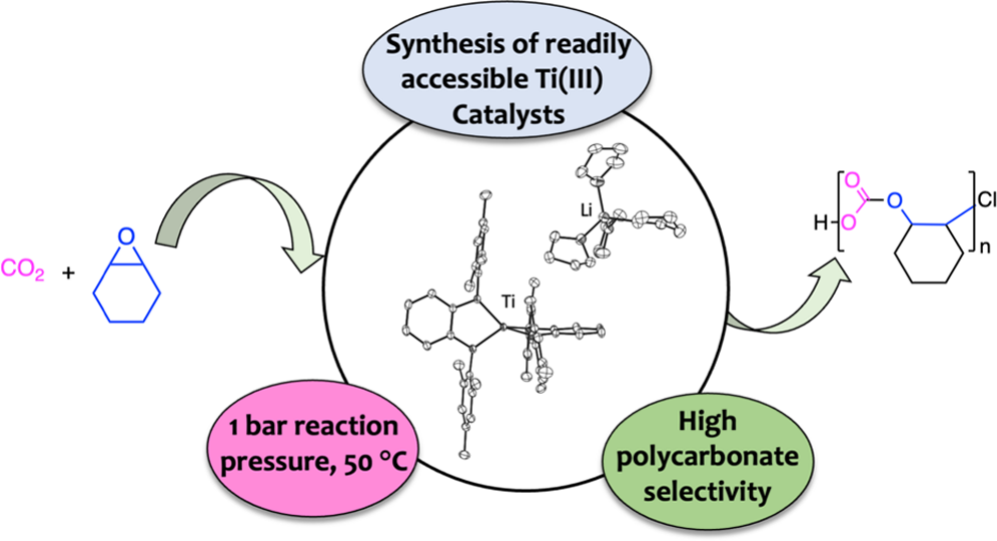

Titanium compounds in low oxidation states are highly reducing species and hence powerful tools for the functionalization of small molecules. However, their potential has not yet been fully realized because harnessing these highly reactive complexes for productive reactivity is generally challenging. Advancing this field, herein we provide a detailed route for the formation of titanium(III) orthophenylendiamido (PDA) species using [LiBHEt_3_] as a reducing agent. Initially, the corresponding lithium PDA compounds [Li_2_(^Ar^PDA)(thf)_3_] (Ar = 2,4,6-trimethylphenyl (^Mes^PDA), 2,6-diisopropylphenyl (^*i*Pr^PDA)) are combined with [TiCl_4_(thf)_2_] to form the heterobimetallic complexes [{TiCl(^Ar^PDA)}(μ-^Ar^PDA){Li(thf)*_n_*}] (*n* = 1, Ar = *i*Pr **3** and *n* = 2, Ar = Mes **4**). Compound **4** evolves to species [Ti(^Mes^PDA)_2_] (**6**) via thermal treatment. In contrast, the transformation of **3** into [Ti(^*i*Pr^PDA)_2_] (**5**) only occurs in the presence of [LiNMe_2_], through a lithium-assisted process, as revealed by density functional theory (DFT). Finally, the Ti(IV) compounds **3**–**6** react with [LiBHEt_3_] to give rise to the Ti(III) species [Li(thf)_4_][Ti(^Ar^PDA)_2_] (Ar = *i*Pr **8**, Mes **9**). These low-valent compounds in combination with [PPN]Cl (PPN = bis(triphenylphosphine)iminium) are proved to be highly selective catalysts for the copolymerization of CO_2_ and cyclohexene epoxide. Reactions occur at 1 bar pressure with activity/selectivity levels similar to Salen–Cr(III) compounds.

## Abstract 2

We describe the synthesis of Ti(III) bis(*o*-phenylendiamido) species, including mechanistic insights gathered by a combination of X-ray studies, NMR spectroscopy, and DFT calculations. In addition, we disclose the application of the titanium(III) compounds as effective catalysts for the selective copolymerization of CO_2_ and cyclohexene epoxide under atmospheric reaction conditions (1 bar).

## Introduction

Low-valent titanium compounds are receiving significant attention due to their versatile applications in organic synthesis, catalysis, and small-molecule activation.^1,2^ However, the chemistry of these reagents is underdeveloped in comparison to mid and late-transition metals, which can be attributed to their strongly reducing character. Therefore, these complexes require powerful stabilizing fragments, typically bulky cyclopentadienyl ligands.^3^ Nevertheless, the use of other supporting fragments has led to new species otherwise not accessible. For instance, Ti(0) and Ti(I) systems are isolated in the form of bisarene species.^3^ In addition, the installation of ligands containing amido fragments such as PNP ([N(2-*i*Pr-4-MeC_6_H_3_)_2_]), amidinate, guanidinate, and β-diketiminate compounds^4^ has enabled access to applications in the field of catalytic dehydrogenation^5^ and hydrogenation^6^ reactions, and more remarkably into the more challenging area of nitrogen fixation.^7^ Comparatively, the use of chelate diamido fragments as ancillary ligands for titanium compounds in low oxidation states has been less explored.

**Figure 1:**
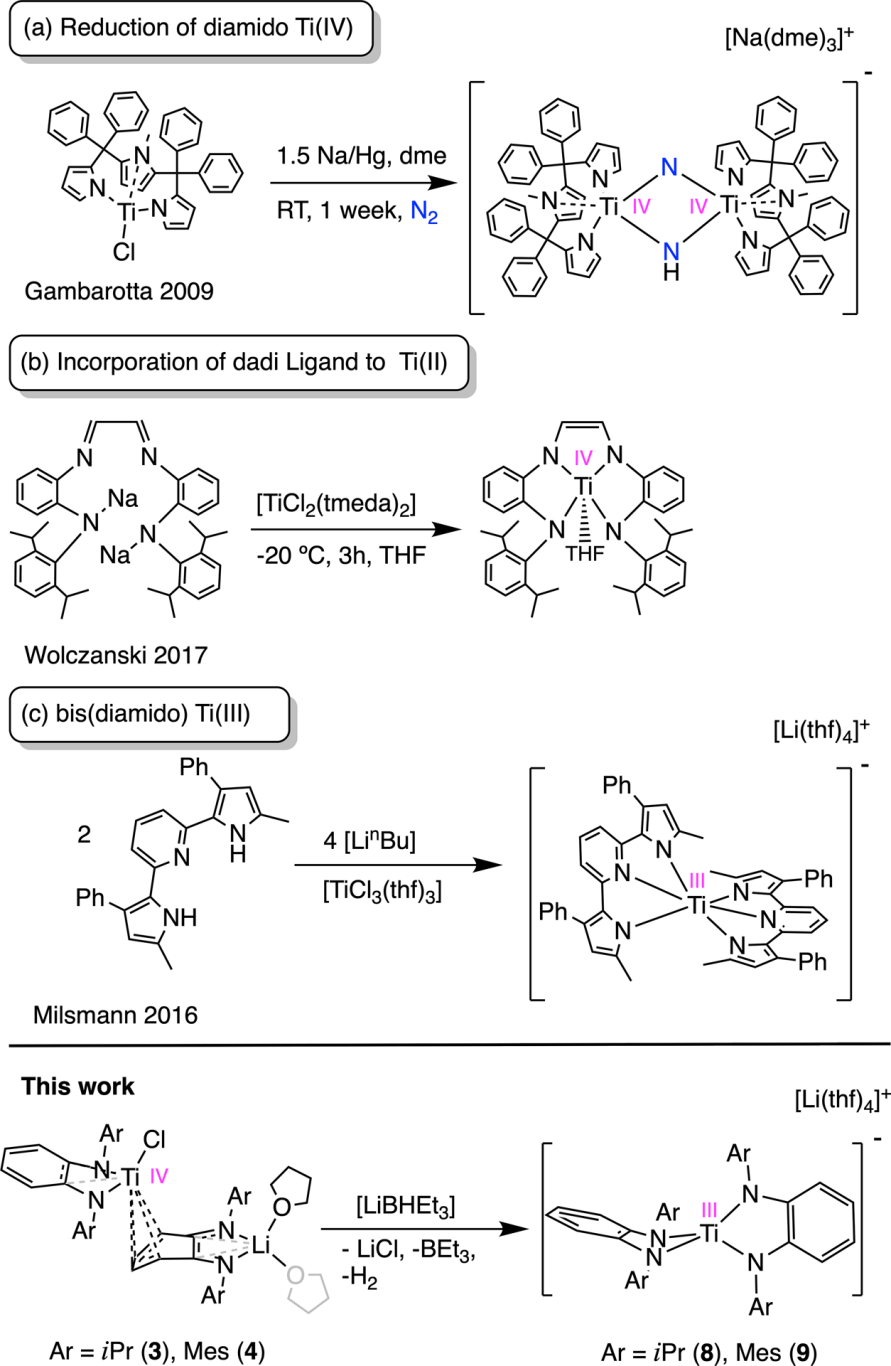
Previous and new work on the preparation of low-valent titanium diamido compounds. (a) Reduction of a tripyrrole Ti(IV) species. (b) Incorporation of the diamido-diimine, dadi, ligand to a Ti(II) precursor. (c) Installation of a 2,6-bis(pyrrolyl)pyridine ligand to the Ti(III) precursor [TiCl_3_(thf)_3_].

Using a tripyrrole dianion, Gambarotta^8^ described the chemical reduction of the corresponding titanium chloride complex with Na/Hg in the N_2_ atmosphere (Figure 1a). Although the reduced species was not isolated, the low valency was evidenced by the splitting and partial hydrogenation of dinitrogen. Later, Wolczanski^9^ explored the incorporation of the diamido fragment present in the ligand diamido-diimine dadi (dadi = {−CH=N(1,2-C_6_H_4_)N(2,6-*i*Pr_2_-C_6_H_3_)}_2_) to the titanium(II) precursor [TiCl_2_(tmeda)_2_] (Figure 1b). Instead of forming the corresponding Ti(II) species, the reaction resulted in the chemical reduction of the diimine fragment and generation of the bis(diamido) Ti(IV) compound [Ti(dadi)(thf)].^9^ For the oxidation state III, Milsmann^10^ reported the isolation of a bimetallic Li/Ti(III) compound upon the reaction of lithium 2,6-bis(pyrrolyl)pyridine with the already reduced [TiCl_3_(thf)_3_], but no further reactivity was studied (Figure 1c).

**Figure 2:**
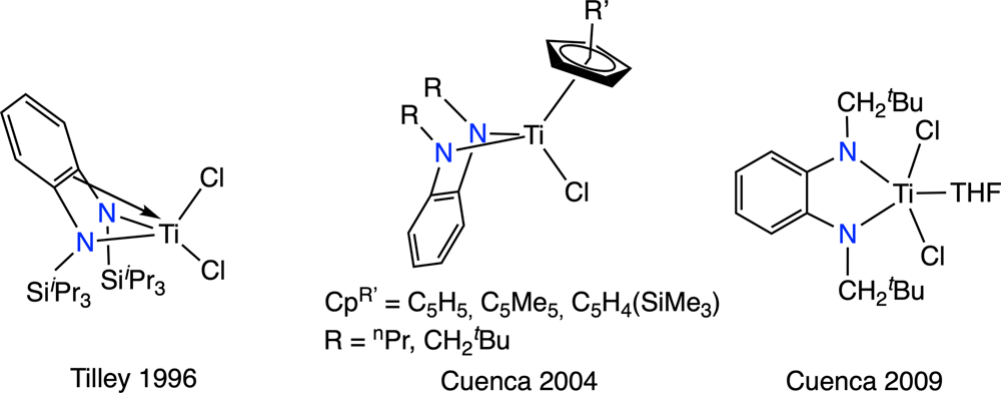
Prior examples of PDA-Ti(IV) compounds.

Among the diamido ligands, ortho-phenylenediamido species (PDA) have been demonstrated to be excellent supporting ligands for strongly reducing species such as Mg(I),^11^ Zn(I), and Ga(II).^12^ In contrast, within the chemistry of titanium, these ligands have been only employed in the preparation of Ti(IV) compounds, where the most used fragments are the *N*,*N*′-disilyl,^13^*N*,*N*′-bis(neopentyl),^14^ and *N*,*N*′-bis(*n*-propyl)^14c,14d^ substituted (Figure 2).

**Figure 3:**
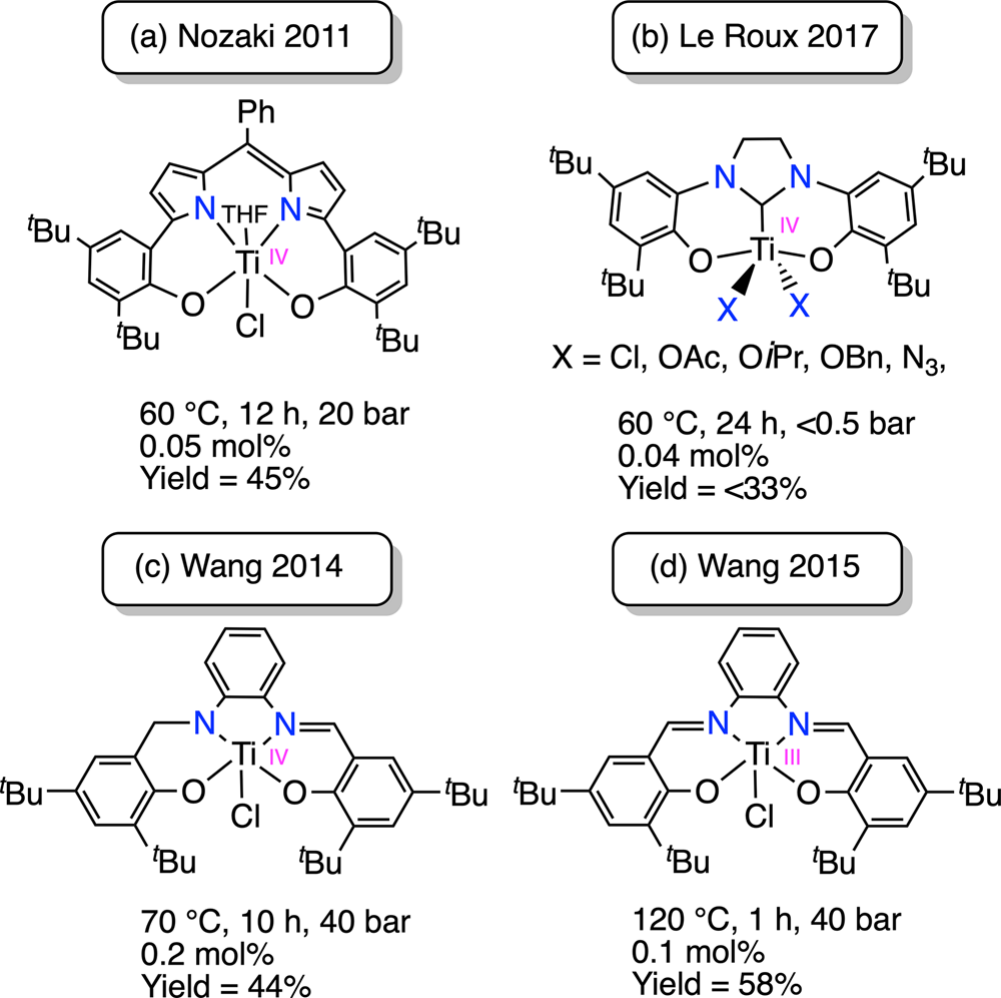
Titanium-based compounds reported for the copolymerization of CO_2_ and CHO.

Titanium-based compounds are particularly attractive catalysts for CO_2_ functionalization through ring-opening copolymerization (ROCOP) with epoxides due to their high abundance, low cost, and limited toxicity.^15−17^ However, the application of these metal complexes has received limited attention compared with species based on Zn(II), Co(II/III), Cr(III), and Al(III).^18^ Despite the recent emergence of Ti(III) species as an efficient catalyst,^19^ this field remains dominated by titanium catalysts in the highest oxidation state.^20^ Revealing the potential of titanium(IV) compounds in this field, Nozaki^21^ reported the [(Boxdipy)TiCl] (Boxdipy = 1,9-bis(2-oxidophenyl)dipyrrinate) (Figure 3a) complex, which in conjunction with [PPN]Cl produces a completely alternating polycyclohexenecarbonate in a 45% yield. The copolymerization reaction involves cyclohexene oxide (CHO), CO_2_ (20 bar), and 0.05 mol % of catalyst and is carried out at 60 °C for 12 h. More recently, Le Roux^22^ described a series of bis-aryloxy N-heterocyclic carbene (NHC) titanium compounds (Figure 3b). These species, at 0.04 mol % catalyst loading, combined with [PPN]Cl mediate copolymerization of CHO with CO_2_ at 60 °C and lower reaction pressure (<0.5 bar). Despite this improvement, the catalytic reaction requires longer reaction times (24 h) and results in low yields (<33%). Using Salen ligands, Wang^23^ developed a [(Salen)Ti(IV)Cl_2_] species that, despite being unable to mediate copolymerization of CHO/CO_2_, selectively generates cyclic carbonate. When the asymmetric Salalen ligand was employed, the catalytic system formed by [(Salalen)TiCl] (Figure 3c) (0.2 mol %) and [PPN]Cl mediates copolymerization of CHO/CO_2_ in a 44% yield, at 70 °C, 40 bar and for 10 h.^23^ Remarkably, the same Wang^19^ reported a more active catalytic system based on Ti(III). The [(Salen)Ti(III)Cl] (Figure 3d) complex in 0.1 mol %, along with [PPN]X (X = Cl, Br, 2,4-dinitrophenolate) salts as cocatalyst, catalyzes the formation of polycyclohexenecarbonate with a 58% yield, requiring 1 h at 120 °C and 40 bar. Notably, this low-valent titanium system mimics the remarkably active and selective Salen–chromium [(Salen)Cr(III)N_3_]/[PPN]Cl binary system, which generates polycyclohexenecarbonate in 85% yield, using 0.04 mol % at 80 °C, 55 bar in 4 h.^24^ The greater catalytic activity of the Ti(III) compound compared with the Ti(IV) compound is rationalized based on the stronger polarity of the Ti(III)–O bond, which favors the reversible formation and dissociation of the Ti–O bonds necessary for the propagation step.^19^ Despite this advance, it is surprising that, to the best of our knowledge, there have not been further reports using a Ti(III) catalyst for the copolymerization of CO_2_ and epoxides. Only Le Roux^22^ attempted to prepare an NHC-based Ti(III) compound as a potential precursor for the copolymerization reaction, albeit the employed ligands proved to be resistant to accommodate the Ti(III).

Bearing in mind the capability of PDA ligands to stabilize low-valent metallic compounds, and the potential of Ti(III) species in the functionalization of CO_2_, herein we describe the synthesis and reduction of bis-PDA Ti(IV) species. The isolated bis(diamido) Ti(III) compounds are characterized by X-ray data and EPR spectroscopy. In addition, DFT calculations were performed in order to fully understand the bonding situation, electronic structure, and the thermodynamics controlling the formation of some of the Ti(IV) precursors of the Ti(III) compounds. The catalytic potential of the Ti(III) species is probed for the copolymerization of CO_2_ and cyclohexene epoxide, generating selective polycarbonate under mild reaction conditions (*p*CO_2_ = 1 bar, 50 °C). Remarkably, compound [Li(thf)_4_][Ti(^Mes^PDA)_2_] **9** displays activity and selectivity levels comparable to Salen–chromium catalysts.

## Results and Discussion

### Synthesis of Ti Compounds

**Figure 4:**
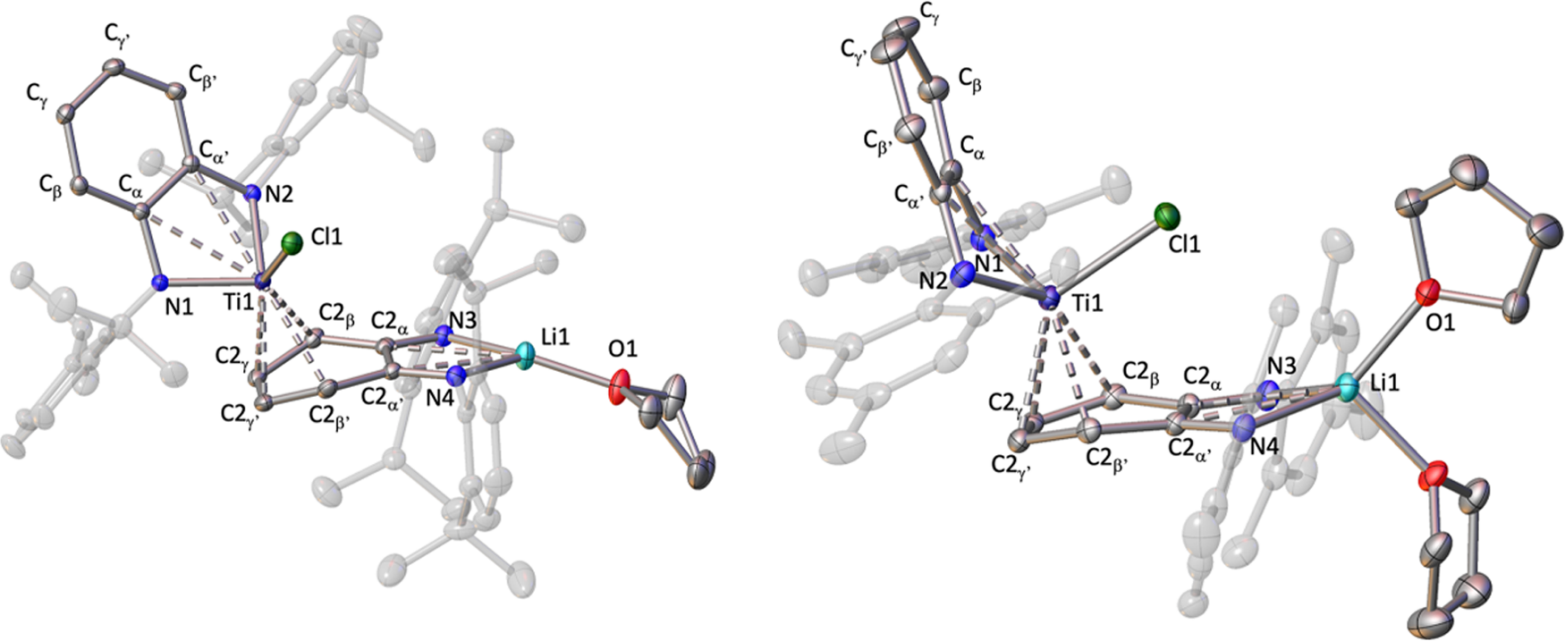
Solid-state structure of compounds **3** (left) and **4** (right) with thermal ellipsoids at 30% of probability. Hydrogens are omitted for clarity. The torsion angle for cent1–cent2–Ti1–Cl1 is 58.98(1)° for **3** and 1.61(3)° for **4**, where cent1 = centroid of diazametallacycle LiN_2_C_2_ and cent2 = centroid of the C2_α_-C2_α′_ ring. The dihedral angle between planes formed by C2_β_–C2_α_–C2_α′_–C2_β′_ and C2_β_–C2_γ_–C2_γ′_–C2_β′_ is 21.1(1)° for **3** and 14.4(3)° for **4**.

**Scheme 1:**
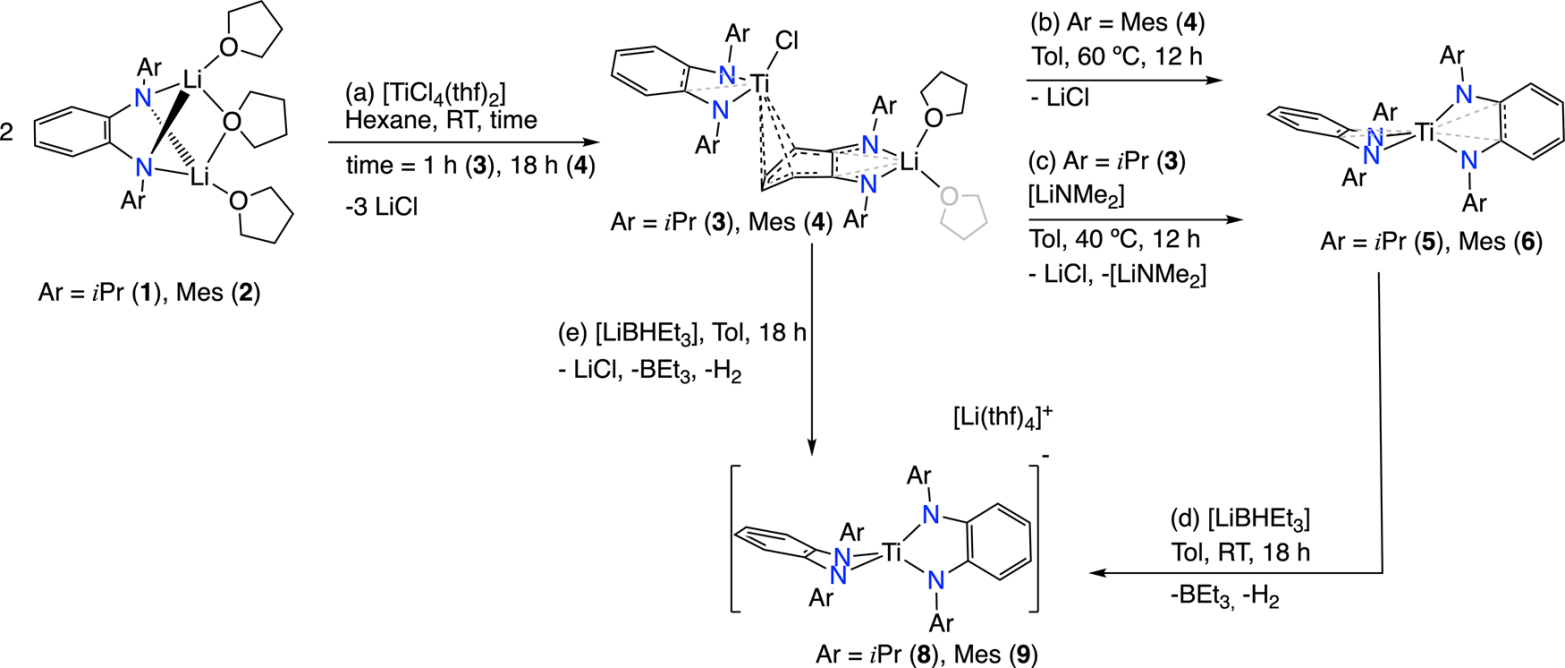
Synthesis of PDA-Titanium Compounds (a) **3** and **4**, (b) **6**, (c) **5**, and (d, e) **8** and **9**.

We began our studies by looking into the incorporation of two equivalents of the mesityl (^Mes^PDA)- and 2,6-diisopropylphenyl (^*i*Pr^PDA)-substituted PDA^2–^ fragments into Ti(III) through transmetalation reaction between the corresponding lithiated precursors **1** and **2** and [TiCl_3_(thf)_3_] in C_6_H_6_. Unexpectedly, analysis of the reaction mixture by ^1^H NMR spectroscopy in C_6_D_6_ revealed the generation of diamagnetic products, which are assigned to compounds **5** and **6** (Figures S14 and S15). A disproportionation reaction is more likely to be responsible for the formation of the Ti(IV) complexes (**5** and **6**). Alternatively, we sought the synthesis of the titanium(IV) precursors and subsequent reduction. Initially, we reacted two equivalents of the ligands ^Ar^PDAH_2_ (Ar = Mes, *i*Pr) with [Ti(CH_2_Ph)_4_] in C_6_D_6_ at temperatures ranging from room temperature to 110 °C. This method was unsuccessful, resulting in no reaction in the case of the bulkier ^*i*Pr^PDAH_2_, while for the ^Mes^PDAH_2_ ligand only small amounts of a compound identified as **6** were formed. Consequently, we explored a second route that involves a transmetalation reaction using the lithium derivatives **1** and **2** and the [TiCl_4_(thf)_2_] starting material (Scheme 1a). The highest yields were achieved using hexane as a solvent. However, the distinct solubility of the final products **3** (soluble) and **4** (insoluble) in this apolar solvent leads to different reaction times, requiring 1 h for **3** and 18 h for **4**. ^1^H NMR spectra of the resulting compounds **3** and **4** in C_6_D_6_ reveal the existence of two chemically distinct PDA fragments, in which one of the ligands displays two resonances for the phenylene fragment at high field (range 5.30–6.41 ppm), indicative of a π-coordination to a metallic center. In addition, an inspection of the reaction mixtures by ^7^Li NMR spectroscopy shows signals at 2.09 and 2.22 ppm for **3** and **4**, respectively. X-ray analysis of single crystals of these compounds reveals partial transmetalation, forming a heterobimetallic compound consisting of a [Li(^Ar^PDA)(thf)*_n_*] (*n* = 1, Ar = *i*Pr; *n* = 2, Ar = Mes) fragment, which binds through the phenylene backbone in a η^4^-C_6_H_4_ fashion to a [TiCl(^Ar^PDA)] moiety (Figure 4).

Structurally, compounds **3** and **4** are similar, although they exhibit different relative dispositions of both fragments reflected by the significantly distinct torsion angle for cent1–cent2–Ti1–Cl1 (see torsion angles in Figure 4). This difference is most likely due to the bulkier nature of the ^*i*Pr^PDA ligands in compound **3** that impedes a close approximation of both PDA fragments as observed in **4** (see van der Waals model representation in Figure S16). In addition, this situation leads to much longer distances between the chlorine and lithium atoms of the vicinal fragments in compound **3** (5.785(3) Å) than the one registered in complex **4** (3.909(6) Å).

Further analysis of the titanium-PDA fragment discloses that the metal in compounds **3** and **4** coordinates with the two nitrogen atoms of the attached ligand, the chlorine atom, and the η^4^-C_6_H_4_ fragment. The latter coordination is confirmed by the puckering of the phenylene ring (see dihedral angles in Figure 4) and the short Ti–C bond distances ranging from 2.281(2) to 2.463(2) Å. Additionally, in both cases, titanium displays an interaction with the electron π density on the C_α_=C_α′_ fragment of the PDA ligand,^13,25^ consistent with the elongation of the latter bond (1.425(2) Å in **3**; 1.427(5) Å in **4**) and the Ti–C bond distances exhibiting an average value of 2.644(3) Å for **3** and 2.548(5) Å in **4**.

Doubly deprotonated PDA species can exist as ortho-diamido, ortho-diiminosemiquinonate, and ortho-benzo-quinodiimine fragments through one- and two-electron oxidation processes.^26^ For the PDA ligand coordinating the titanium atom through the nitrogen atoms (henceforth PDA-N) both X-ray and optimized DFT geometries (at B3LYP-D3BJ(SMD)/def2SVP level of theory) show a notable degree of bond length equalization for the C–C bonds (average 1.39(1) Å), and characteristic single C–N bond lengths (Table 1), pointing to a diamido nature for this fragment.^27^ Further support for this diamido character is found in the Ti–N bonds of **3** (average = 1.95(1) Å) and **4** (average 1.928(3) Å), which lie in the range for reported Ti(IV) compounds supported by diamido ligands.^13,14c,25a,25c,28^

**Table 1:** X-Ray and Optimized DFT (B3LYP-D3BJ/Def2SVP Level of Theory) Bond Lengths (in Å), and Bond Orders for the Phenyl Ring of PDA-N in Compounds **3** and **4**

	Compound **3**			Compound **4**		
	Bond length			Bond length		
Bond	X-ray	DFT	Bond order	X-ray	DFT	Bond order
C_α_–N^a^	1.404(7)	1.397	1.05	1.406(1)	1.398	1.08
C_α_–C_α′_	1.425(2)	1.432	1.13	1.427(5)	1.432	1.14
C_α_–C_β_^a^	1.399(1)	1.407	1.25	1.403(1)	1.406	1.26
C_β_–C_γ_^a^	1.380(2)	1.392	1.40	1.373(1)	1.392	1.40
C_γ_–C_γ′_	1.384(3)	1.404	1.34	1.388(6)	1.404	1.35

a Bond distance average.

Contrary to PDA-N, the PDA ligand bound to lithium and coordinating titanium through the phenylene ring (henceforth PDA-Ring) displays experimental and DFT-calculated shorter average C–N bonds, as well as the loss of the bond length equalization of the phenyl ring (Table 2). Additionally, and in agreement with the puckering of the rings, the phenyl moieties have lost their planarity.

**Table 2:** X-Ray and Optimized DFT (B3LYP-D3BJ/Def2SVP Level of Theory) Bond Lengths (in Å), and Bond Orders for the Phenyl Ring of PDA-Ring in Compounds **3** and **4**

	Compound **3**			Compound **4**		
	Bond distance			Bond distance		
Bond	X-ray	DFT	Bond order	X-ray	DFT	Bond order
C_2α_–N	1.313(3)	1.320	1.24	1.315(3)	1.326	1.28
C_2α_–C_2α′_	1.479(2)	1.489	0.95	1.488(4)	1.487	0.98
C_2α_–C_2β_^a^	1.431(1)	1.431	1.17	1.432(1)	1.429	1.19
C_2β_–C_2γ_^a^	1.415(2)	1.419	1.25	1.416(2)	1.418	1.26
C_2γ_–C_2γ′_	1.385(3)	1.399	1.32	1.389(5)	1.399	1.33

a Bond distance average.

A structurally similar heterobimetallic Li/Ta PDA-based complex has been described by Song.^29^ According to the metrical data, the author proposes a diiminocyclohex-2-ene-1,4-diide as the predominant resonant form for the PDA ligand. Based on the electropositive nature of the tantalum atom, they describe the interaction between the [TaClMe_3_] moiety and the phenylene ring of the [Li(OEt_2_)(^*i*Pr^PDA)(thf)] fragment as a metallacyclopentene. In our case, the shorter C2_γ_–C2_γ′_ bonds (1.385(3) Å for **3** and 1.389(5) Å for **4**) than those for C2_β_–C2_γ_ (average 1.415(2) Å for **3**; 1.416(2) Å for **4**) favor a metallacyclopentene interpretation, whereas the significantly longer Ti–C_β_ bonds (average 2.44(2) Å for **3**; 2.435(1) Å for **4**) than the Ti–C_γ_ distances (average 2.29(1) Å for **3**; 2.302 (7) Å for **4**) are contrary to this interpretation. Aiming to clarify the bonding situation, we analyzed the electronic structure of **3** and **4** by means of DFT calculations using two parameters: oxidation states (OS) and bond orders (B3LYP-D3BJ/def2TZVP//B3LYP-D3BJ(SMD)/def2SVP level of theory).

First, the OS of Ti and the formal charge of ligands for **3** and **4** were assigned using the effective oxidation state (EOS) approach.^30−32^ The OS of titanium and both diamido (PDA) units are, respectively, +4 and −2. Considering the occupation numbers of the last occupied spin-resolved effective fragment orbital (EFO) and the first unoccupied EFO (see Tables S1 and S2), this OS assignation in **3** and **4** is indisputable. These results suggest that no redox reactions have taken place, and hence the diiminosemiquinonate and benzo-quinodiimine forms are unlikely to define the PDA-Ring unit.

The Ti–C bond orders in the Ti–arene interaction for compounds **3** and **4** reveal very similar values between Ti and the four carbons C_β_, C_γ_, C_β′_, and C_γ′_ (Table 3). Regarding the PDA-Ring fragment, the C–N bond orders lie between single and double bond characters, and compared with PDA-N, the C_α_–C_β_ and C_β_–C_γ_ bond orders reveal a decrease.

**Table 3:** Bond Lengths (in Å) and Bond Order for Each Ti–C Bond of the Phenyl Ring of PDA-Ring in Compounds **3** and **4**

	Compound **3**			Compound **4**		
	Bond distance			Bond distance		
Atom	X-ray	DFT	Bond order	X-ray	DFT	Bond order
C_α_^a^	3.00(2)	2.88	0.077	2.883(6)	2.84	0.085
C_β_	2.416(2)	2.41	0.263	2.437(3)	2.43	0.251
C_γ_	2.281(2)	2.31	0.263	2.308(3)	2.32	0.256
C_γ′_	2.307(2)	2.32	0.257	2.295(3)	2.32	0.255
C_β′_	2.463(2)	2.48	0.241	2.434(3)	2.44	0.241

a Bond distance average.

**Figure 5:**
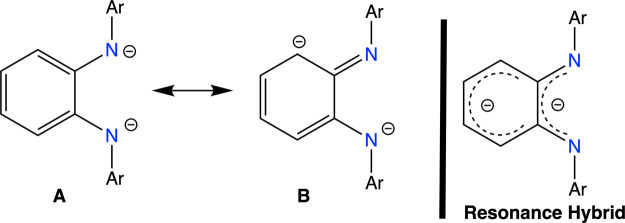
Resonance forms and resonance hybrid for the PDA^2–^ ligand.

Overall these data suggest that the coordination of the PDA-Ring to Ti(IV) is better described by a Ti-η^4^-arene, where the phenylene ring is acting as an anionic π-electron-donating ligand according to the resonance form **B** and its resonance hybrid shown in Figure 5.

Supporting this bonding mode, the registered Ti–C bond distances for compounds **3** and **4** (Table 2) are reminiscent of previously reported Ti–arene compounds, in which Ti-(η^4^-arene) coordination is observed.^33^ For example, the structurally characterized titanium-anthracene^33a^ complexes [Ti(η^6^-C_14_H_10_)(η^4^-C_14_H_10_)(η^2^-dmpe)], [Ti(η^4^-C_14_H_10_)(η^2^-C_14_H_10_)(η^5^-C_5_Me_5_)]^−^ (Ti–C range = 2.295(3)–2.424(4) Å), and Ti-naphtalene [Ti(η^4^-C_10_H_8_)_2_(SnMe_3_)_2_]^2–^ (Ti–C range = 2.30(1)–2.34(1) Å).^33b^ Additionally, these reports also describe longer distances for the C_β_–C_γ_ (range 1.427(6)–1.44(2) Å) compared with the C_γ_–C_γ′_ (1.375(6)–1.38(2) Å) for the aromatic ring bonded to the titanium atom.^33^

**Figure 6:**
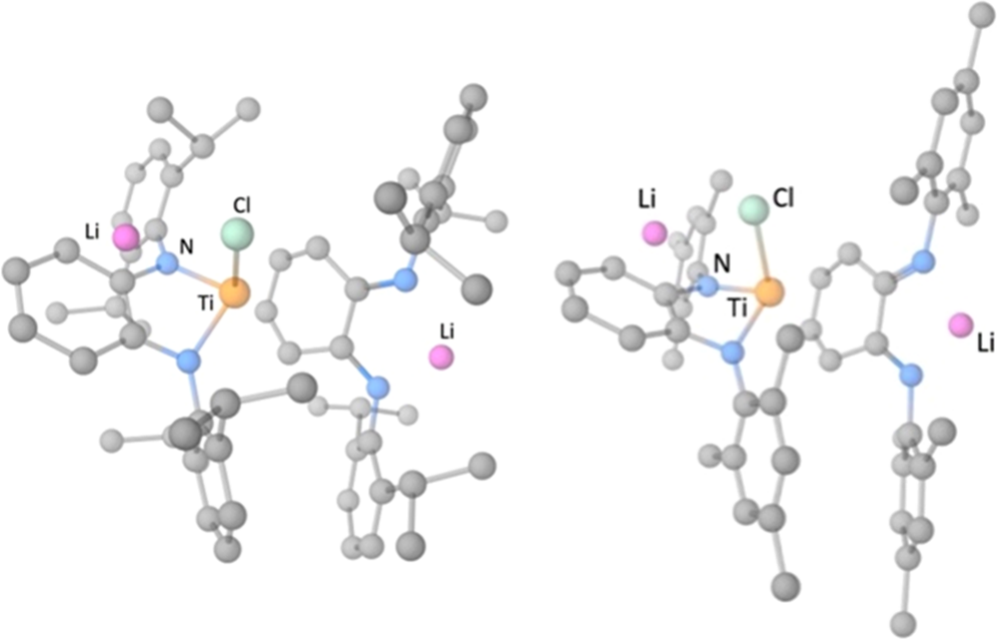
Geometries for the reactant complex including a second lithium cation, for compounds **3** (left) and **4** (right). The hydrogen atoms are hidden for clarity.

Next, we studied the potential transformation of species **3** and **4** toward the desired titanium(IV) bis(diamido) precursors. In agreement with the shorter Li···Cl distance found for compound **4** compared with **3**, the former facilitates LiCl release upon heating at 60 °C, generating compound **6** (Scheme 1b). In contrast, intermediate **3** proves to be thermally robust, as no evolution is detected upon thermal treatment. The observed differences in reactivity for compounds **3** and **4** can be related to the exergonicity of the reactions computed with DFT (see Table S3). Thus, the formation of **6** from **4** is exergonic (Δ*G*^0^ = −2.1 kcal/mol, see Figure S1 and Table S3), whereas the equivalent reaction to give rise to **5** from **3**, with the bulkier *i*Pr ligand, is strongly endergonic (Δ*G*^0^ = + 12.8 kcal/mol, see Figure S3 and Table S3). Therefore, while the formation of **6** is thermodynamically favorable, the generation of **5** is not favorable, explaining why **3** does not evolve to the desired bis(amido) titanium species **5** by heating. Remarkably, if the thermodynamics of the transformations of **3** (**4**) to **5** (**6**) are simulated including a second lithium cation in the reactant complex (**3** or **4**), a noteworthy observation emerges. Through the interaction of lithium with the nitrogen atoms of PDA-N and the chloride (Figure 6), the complete transmetalation is strongly exergonic for **4** (Δ*G*^0^ = −17.2 kcal/mol, see Figure S2 and Table S3) and is isoergonic for **3** (Δ*G*^0^ = + 0.7 kcal/mol, see Figure S4 and Table S3). Thus, the inclusion of a second Li^+^ in the reactant complex strongly contributes to decreasing the reaction Gibbs energy for the formation of the bis(amido) titanium species **5** and **6**. In agreement with these DFT results, the reaction of a toluene solution of species **3** with 1 equiv of the lithium amide [Li(NMe_2_)] at 40 °C leads to the isolation of compound **5** in a 67% yield along with the recovering of the employed lithium amide (Scheme 1c).

**Figure 7:**
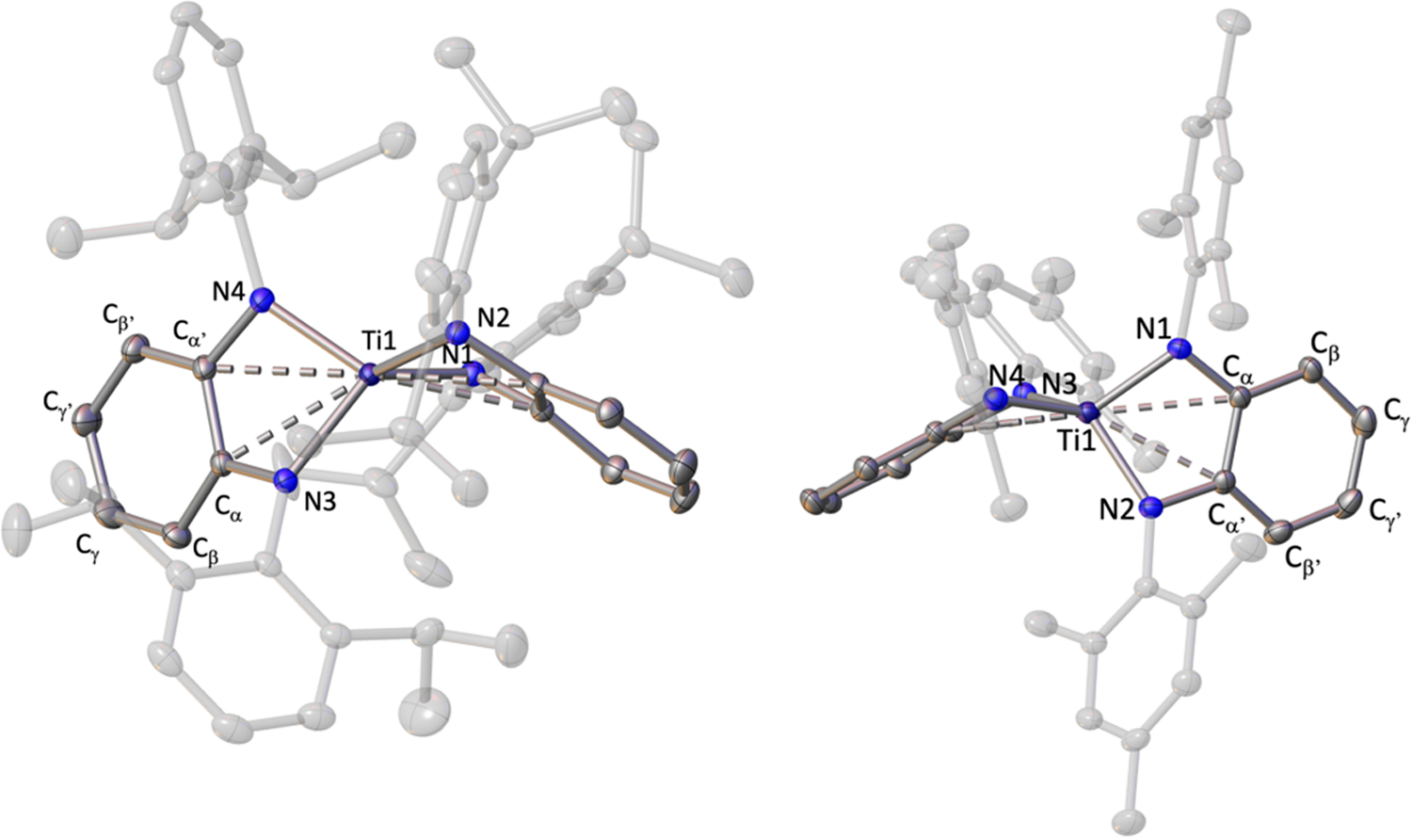
Solid-state structure of compounds **5** (left) and **6** (right) with thermal ellipsoids at 30% of probability. Hydrogens are omitted for clarity.

The solid-state structures of compounds **5** and **6** (Figure 7) show the titanium atoms coordinating the two ^Ar^PDA ligands in a σ^2^, π fashion via four Ti–N bonds (average values: 1.94(3) Å for **5** and 1.92(3) Å for **6**) and by interaction with the C_α_=C_α′_ fragment, according to the lengthening of this bond (1.420(1) Å for **5** and 1.436(1) Å for **6**) and the average distances between titanium and the carbon atoms (2.54(1) Å in **5** and 2.57(1) Å in **6**). These geometrical parameters resemble those registered for the titanium-PDA-N fragment in compounds **3** and **4**, and the homoleptic bis(diamido) titanium compounds bearing the *N*,*N*′-bis(2,6-diisopropylphenyl)-1,4-diazabutadiene^25a^ and the 1,2-bis[(2,6-diisopropylphenyl)imino]acenaphthene ligands displaying a similar σ^2^, π coordination.^34^

Consistent with the diamido nature of the PDA ligands, the average C–N bond lengths are 1.4054(9) Å for **5** and 1.411(2) Å for **6**. Moreover, the phenylene ring retains the aromaticity, displaying C–C average distances of 1.38(1) Å for **5** and 1.387(9) Å for **6**, excluding the longer C_α_=C_α_′ bonds. To relieve the steric congestion created by the two ^Ar^PDA ligands, they are arranged in a staggered disposition displaying a dihedral angle between the planes formed by the PDA units of 61.94(5)° for **5** and 69.04(9)° for **6**. In addition, the wingtip aryl substituents adopt a nearly orthogonal disposition relative to the central phenylene fragments with dihedral angles ranging from 67.84(8) to 74.11(8)° for compound **5** and from 73.4(1) to 80.7(1)° for compound **6**. This situation is reflected in the ^1^H NMR spectrum of **5** in C_6_D_6_, which shows four sets of signals for the isopropyl and phenylene groups. In contrast, compound **6** displays in its ^1^H NMR spectrum in C_6_D_6_ one set of broad signals for the methyl substituents, which can be attributed to a rapid rotation around the N–C(Ar) bonds on the NMR timescale. In agreement with the latter, the ^1^H NMR of compound **6** in C_7_D_8_ at 233K shows the inequivalence of the methyl groups (Figure S37).

**Scheme 2:**
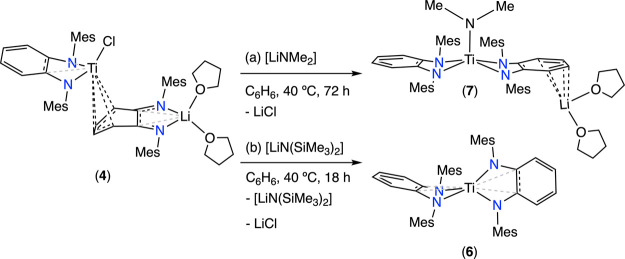
Synthesis of Compound **7**

Contrasting with the exclusive transformation of **3** into **5** in the presence of [LiNMe_2_], when we reinvestigated the synthesis of **6** by heating a toluene solution of **4** assisted by [LiNMe_2_], a mixture of compound **6** and a new species was observed. Longer reaction times (Scheme 2a) resulted in the consumption of **6** in favor of the new product, which incorporates an anionic [NMe_2_]^–^ fragment, according to the signal observed at 3.19 ppm by ^1^H NMR spectrum.

**Figure 8:**
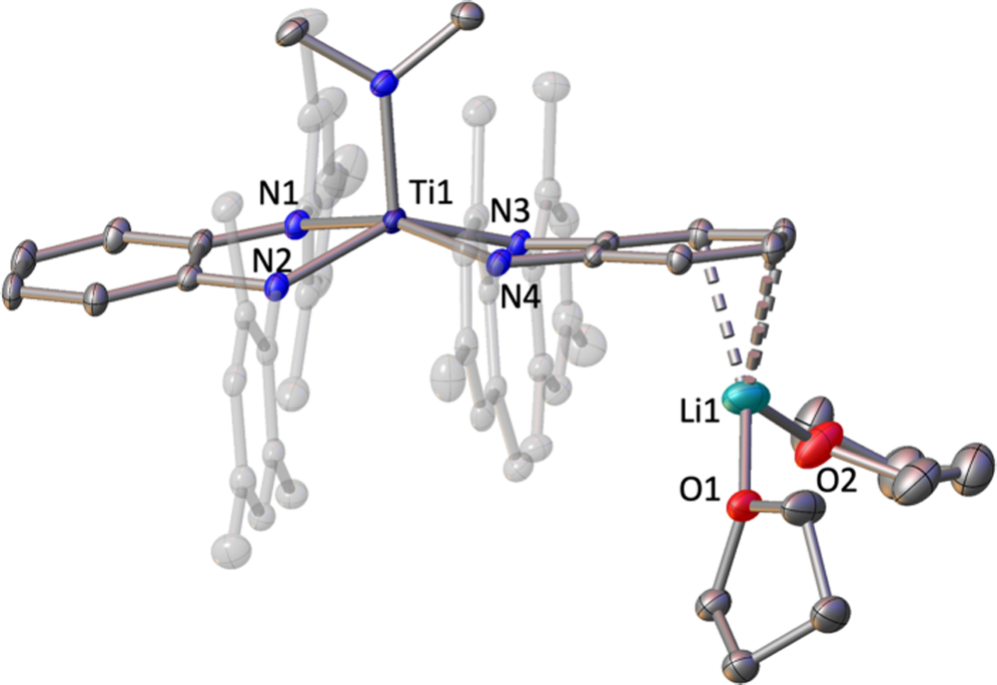
Solid-state structure of compound **7** with thermal ellipsoids at 30% of probability. Hydrogens are omitted for clarity.

X-ray analysis of single crystals grown by slow evaporation in benzene reveals the formation of the contacted ion-paired species [Li(thf)_4_][Ti(^Mes^PDA)_2_(NMe_2_)] (**7**) (Figure 8), in which the cationic fragment [Li(thf)_2_] is bounded through a phenylene unit of one PDA ligand.

The solid-state structure reveals how the PDA units around the titanium atom are capable to transition from the original pseudo-tetrahedral geometry, found in the bis(diamido) compound **6**, to a relative pseudo-planar disposition (dihedral angle between the PDA planes is 12.18(8)°). This rearrangement of the PDA fragments results in elongated Ti–N bonds (Δ*d*_average_ Ti–N = 0.15 Å compared with **6**) and Ti···C_α,α′_ distances (Δ*d*_average_ Ti···C = 0.34 Å compared with **6**) to accommodate a fifth amido group. The molecular structure resembles those reported by Wolzcanski for a series of ionic compounds supported by the bis(diamido) dadi^4–^ ligand with a general formula [Li(thf)_2.5–4_][Ti(dadi)X] (X = Me, O*i*Pr, H).^9^ In our case, the PDA ligands display average C–N and C–C bond distances of 1.39(1) and 1.39(1) Å, respectively, in agreement with the diamide form. The titanium atom is shifted out of the N_4_ plane by 0.474(1) Å and forms the shortest Ti–N bond (1.909(3) Å) with the NMe_2_ unit.

Repeating the reaction between compound **4** and the bulkier reagent [LiN(SiMe_3_)_2_] only leads to the formation of the final product **6** (Scheme 2b). This result along with the lack of incorporation of an anionic [NMe_2_]^−^ into the sterically congested species **5** suggests that the formation of ionic compounds similar to **7** is ruled by the balance of steric properties between the lateral substituents of the PDA ligands and the incoming anionic fragment.

### Reduction of the Titanium(IV) Compounds

The observed flexibility of the PDA ligands to accommodate an additional and relatively small fragment of titanium encouraged us to explore the formation of a possible titanium hydride species as a potential pathway for the chemical reduction of titanium via hydrogen release, similar to previous reports.^35^ Accordingly, the reaction of **3** and **4** or **5** and **6** with [LiBHEt_3_] generates in a straight manner the heterobimetallic Li/Ti(III) species **8** and **9** (Scheme 1d,e). It is reasonable to argue that starting from compounds **3** and **4**, they are first transformed into **5** and **6** assisted by the presence of the second lithium reagent. Subsequently, the reduction of Ti(IV) proceeds via initial hydride transfer from boron to titanium releasing BEt_3_ (detected by ^1^H NMR). This process leads to the formation of an ionic titanium hydride species “[Li(thf)_4_][TiH(^Ar^PDA)_2_]”, akin to the isolated species **7**. In the last step, the putative titanium hydride compound evolves toward the Ti(III) species and produces molecular H_2_. The H_2_ equivalents produced during the reaction time at ambient temperature in THF were determined by monitoring the pressure variation in a closed reaction vessel and using the Man on the Moon X102 device.^36^

Compounds **8** and **9** are paramagnetic with a d^1^ configuration according to their EPR spectra. At a temperature of 77 K in THF, these species exhibit an axial symmetry and *g* values (Figure S5; *g*_⊥_ = 1.978 and *g*_||_ = 1.950 for **8**; *g*_⊥_ = 1.972, and *g*_||_ = 1.935 for **9**) similar to the previously reported Ti(III) [(NacNac)Ti(CH_2_^*t*^Bu)_2_] species.^37^

**Figure 9:**
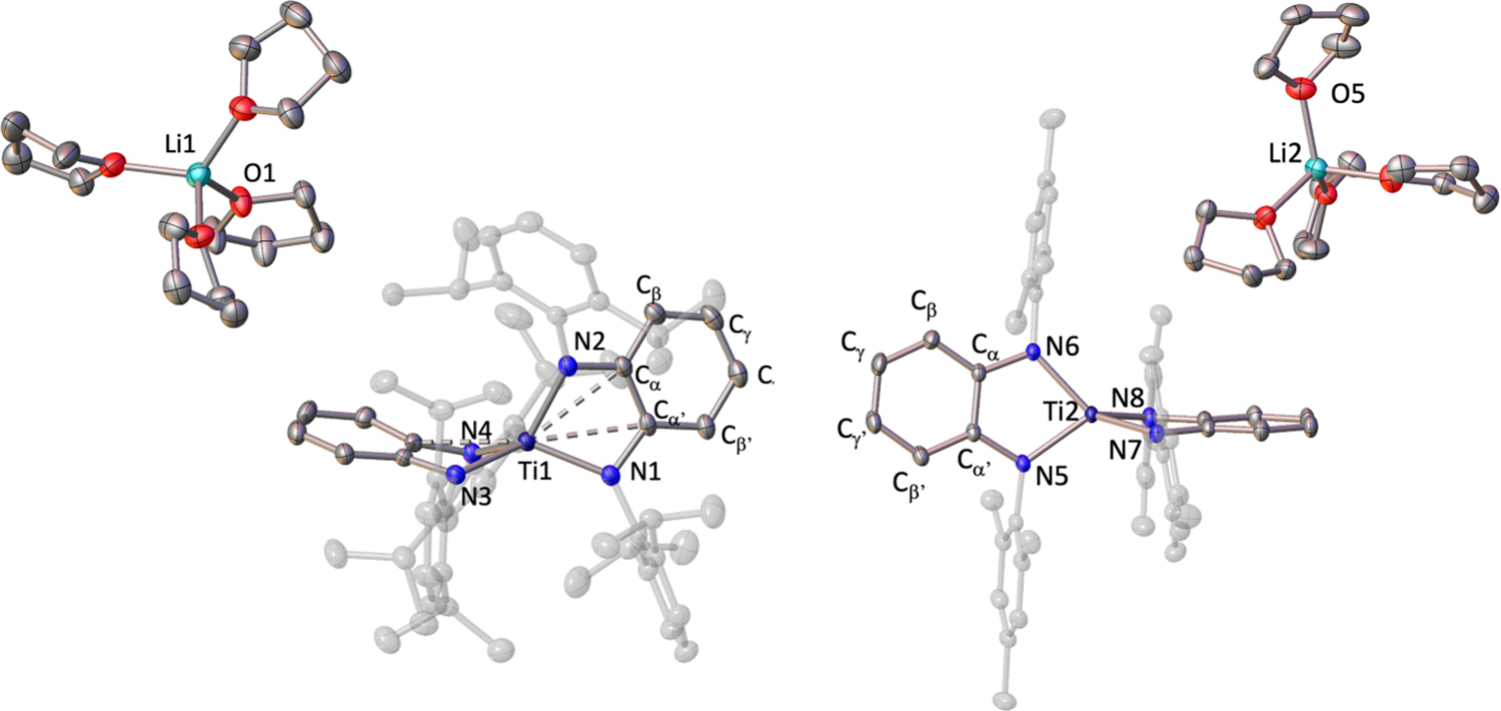
Solid-state structure of compounds **8** (left) and **9** (right) with thermal ellipsoids at 30% of probability. Hydrogens are omitted for clarity. Only one independent crystallographic molecule of the two found for compound **9** is shown.

In the solid state, the molecular structures of **8** and **9** (Figure 9) feature a solvent-separated species formed by a cationic [Li(thf)_4_]^+^ fragment and the anionic [Ti(^Ar^PDA)_2_]^−^ (Ar = *i*Pr **8**, Mes **9**) moiety.

The structural data for ^Ar^PDA fragments in **8** and **9** are nearly identical to the data found for the bis(diamido) Ti(IV) precursors **5** and **6**. Thus, the C–N (1.406(2) Å for **8**; 1.405(5) Å for **9**) and C–C (1.39(1) Å for **8**; 1.389(9) Å for **9**) bond distances of compounds **8** and **9** display values analogous to those found for **5** (C–N = 1.4054(9) Å; C–C = 1.38(1) Å) and **6** (C–N = 1.411(2) Å; C–C = 1.387(9) Å), which is also in agreement with the diamido nature of the PDA ligands. The ^Ar^PDA ligands in **8** and **9** are arranged in a staggered relative position with dihedral angles slightly larger (73.42(7)° for **8**; 69.33(6) and 82.37(6)° for **9**)^38^ than those of **5** (61.94(5)°) and **6** (69.04(9)°). Likewise, the distance between Ti and the C_α_=C_α′_ fragments is significantly longer in compounds **8** (2.70(2) Å) and **9** (2.83(5) Å) compared with **5** (2.54(1) Å) and **6** (2.57(1) Å). The lower oxidation state in compounds **8** and **9** is reflected in elongated Ti–N bonds. Thus, the titanium–nitrogen average bond lengths of 2.01(3) Å for **8** and 2.00 (1) Å for **9** are longer than the values observed for compounds **5** (1.94(3) Å) and **6** (1.92(3) Å). In comparison to structurally characterized aryl-amido Ti(III) species, the Ti–N bonds are lengthened by ca. 0.1;^39^ however, they are similar to those reported with the bulkier bis(silyl)amido fragments in [Ti(N(SiMe_3_)_2_)_3_] and solvated species.^40^

### CO_2_/Epoxide Copolymerization

The structurally similar pair of compounds **5**, **8** and **6**, **9** differs in the oxidation state of titanium. Therefore, they offer a great opportunity to investigate the influence of the oxidation state of the metal in the functionalization of CO_2_ via copolymerization with cyclohexene oxide. Since our titanium compounds lack an initiating group, we combined compounds **5**, **6** and **8**, **9** with [PPN]Cl [PPN = bis(triphenylphosphine)iminium] as the source of an anionic chloride. Using a 2.5 mol % of titanium species along with 2.5 mol % of cocatalyst under 1 bar pressure of CO_2_ at room temperature during 18 h reveals modest to good conversion levels (30–66%, Table 4, entries 1–4) with marked differences in selectivity based on the oxidation state.

**Table 4:** Ring-Opening Copolymerization (ROCOP) of CO_2_ and CHO Using Catalysts **5**–**9**/PPNCl^a^

Entry	Catalyst	*T* (°C)	Cat/[PPN]Cl/CHO (mol %)	Conv. (%)^b^	Carbonate linkages (%)^c^	*M*_n_ (kg mol^–1^)^d^	*Đ*_M_^d^
1	**5**	RT	2.5/2.5/100	30	0^e^	ND	ND
2	**6**	RT	2.5/2.5/100	66	0^e^	ND	ND
3	**8**	RT	2.5/2.5/100	41	62	ND	ND
4	**9**	RT	2.5/2.5/100	50	71	ND	ND
5^f^	**9/12-crown-4**	RT	2.5/2.5/100	55	70	ND	ND
6	**9**	RT	2.5/5/100	80	>99	ND	ND
7		RT	0/5/100	0			
8	**9**	RT	0.5/1/100	23	>99	ND	ND
9	**9**	50	0.5/1/100	57	>99	3.9	1.2
10	**9**	70	0.5/1/100	73	ND^g^	ND	ND
11	**9**	50	0.3/0.6/100	53	>99	3.7	1.14
12	**9**	50	0.2/0.4/100	21	>99	3.4	1.19
13	**9**	50	0.1/0.2/100	17	>99	3.1	1.14
14	**[(Salen)Ti(III)Cl]**	50	0.5/1/100	73	56	ND	ND
15	**[(Salen)Cr(III)Cl]**	50	0.5/1/100	30	>99	3.9	1.27

a Reaction conditions: 1 bar CO_2_, 18 h.

b Determined by ^1^H NMR spectroscopy of the crude mixture reaction by comparison of the relative integrals of the resonances assigned to the carbonate (4.65 ppm for the PCHC and 4.00 ppm for *trans*-CHC) and ether (3.45 ppm) linkages against *cis*-CHO (3.00 ppm).

c Determined by ^1^H NMR spectroscopy by comparison of the relative integrals of the resonances due to the polymer (4.65 ppm) and ether (3.45 ppm).

d Determined by GPC in thf, relative to polystyrene standards. For those cases in which oligomers or a mixture of cyclic carbonate and polycarbonate are obtained, *M*_n_ and *Đ*_M_ values were not determined.

e Only the formation of polyether was detected. Therefore, the *M*_n_ and *Đ*_M_ values were not determined.

f 2.5 mol % of 12-crown-4 was added.

g A reliable integral value for polycarbonate and cyclic carbonate could not be obtained due to the close proximity of the signals.

While the Ti(IV) species (**5** and **6**) provide only polyether with no CO_2_ intake (Table 4, entries 1–2), the Ti(III) compounds (**8** and **9**) display the formation of polycarbonate with modest levels of CO_2_ incorporation (Table 4, entries 3–4). The better performance of the ionic compounds **8** and **9** is most likely due to the combination of the electronic saturation of the Ti center bounded to two PDA^2–^ ligands and the lower oxophilic nature of Ti(III). These factors result in more polarized Ti–O bonds compared with those established by the neutral Ti(IV) species, which would favor the insertion of CO_2_ into the Ti–O bond during the propagation step. It is noteworthy to mention that despite the fact that Ti(III) in compounds **8** and **9** are expected to be poor Lewis acids, the required epoxide coordination is concentration favored as the reactions are conducted in neat epoxide. In addition, the anionic compounds **8** and **9** feature a lithium cation that can cooperate with titanium toward the copolymerization reaction. This synergic effect between an alkali metal and a transition metal has been well documented by Williams,^41^ who combining cobalt with alkali metals provides an efficient strategy to promote catalyst performance for the copolymerization of CO_2_ and epoxides. To determine the potential cooperation of lithium in the catalytic reaction, we conducted the copolymerization of CHO/CO_2_ using catalyst **9** in the presence of the 12-crown-4 to block the coordination sites of Li. Adding the crown ether does not have an impact on the catalytic activity (Table 4, entry 5), which suggests that the lithium atom does not play a significant role in the catalytic reaction.

Comparison of entries **3** and **4** displays that compound **9**, with a more accessible Ti(III) center, shows better activity and selectivity than the sterically bulkier **8**, and therefore we continued our studies with species **9**. Based on the well-established fact that an increase of the cocatalyst loading enhances the activity and selectivity,^42^ we increased the catalyst/[PPN]Cl ratio to 1:2, leading to selective (>99%) formation of polycarbonate in an 80% conversion (Table 4, entry 6). Notably, no epoxide conversion was observed when the catalytic reaction was conducted under the optimized conditions without using the titanium catalyst **9** (Table 4, entry 7). Despite the good result obtained in entry 6, isolation of the formed polycarbonate by precipitation proved to be difficult, most likely due to the formation of oligomers. Determined to increase the chain length, we decreased the catalyst loading up to 0.5 mol % while maintaining the 1:2 catalyst/[PPN]Cl ratio. However, it resulted in a drop in conversion to 23% (Table 4, entry 8). The latter was improved by a slight increase in reaction temperature to 50 °C (Table 4, entry 9). In this case, the desired polycarbonate was isolated by precipitation according to a molecular weight of 3.9 kg·mol^–1^ determined by GPC analysis, which also discloses a narrow dispersity (*Đ*_M_ = 1.2). An increase in the reaction temperature enhances the conversion to 73%, but impacts the polycarbonate selectivity, as cyclohexene carbonate is now detected (Table 4, entry 10). Holding the reaction temperature to 50 °C and CO_2_ pressure to 1 bar, our system proved to retain similar levels of activity up to catalyst loading of 0.3 mol % (Table 4, entry 11), generating the desired polycarbonate in 53% conversion, and with comparable molecular weights and dispersity to entry 9. However, a further decrease in the catalyst concentration to 0.2–0.1 mol % (Table 4, entries 12 and 13) leads to a significant decrease in conversion, although the generated polycarbonate shows similar properties (*M*_n_ and *Đ*_M_).

The MALDI-ToF-MS spectrum of the polycarbonate with a greater value of M_n_ (Table 4, entry 9) displays one major series of peaks in accordance with the formula [{HO(CHO-CO_2_)*_n_*OCHC_4_H_8_CHCl}Na]^+^, confirming the role of the chloride anion as an initiator. Furthermore, the MALDI-ToF-MS spectrum also shows two additional series of peaks with the same polycarbonate unit as the previous one, albeit with alkoxide fragments as ending groups instead of the chlorine atom.^43^ Similar results in CO_2_/epoxide copolymerization have been rationalized by chain transfer reactions with organic alcohols generated upon partial hydrolysis of the epoxide.^44^ In our case, GC-MS and ^1^H NMR analysis of cyclohexene epoxide after being exposed to CO_2_ under the reaction conditions employed during catalysis (18 h, 50 °C) did not show the presence of any organic alcohol (Figure S11). Therefore, it is reasonable to argue that the alkoxides initiating the polymerization process are a consequence of minor side reactions of our titanium catalyst with the epoxide, as it has been reported for similar metal-mediated copolymerization processes.^45^

Our PDA-Ti(III) catalyst is one of the rare examples of Ti-based systems that can promote the copolymerization of CHO and CO_2_ at atmospheric pressure.^22,46^ Thus, the series of tridentate NHC–titanium compounds reported by Le Roux^22,46^ catalyze CO_2_/epoxide copolymerization under similar reaction conditions to our system (0.5 bar CO_2_ and 60 °C). Although for the NHC–Ti systems lower conversions (<38%) are reported, they provide polycyclohexanecarbonate with much greater molecular weights (7.4 kg/mol).

In order to benchmark the catalytic activity and selectivity of compound **9**, we conducted the copolymerization of CHO/CO_2_ under the optimized conditions (0.5 mol %, 50 °C, 1 bar, 18 h) with the Ti(III) [(Salen)TiCl] reported by Wang^19^ and the homolog [(Salen)Cr(III)Cl]^47^ complex. Comparison with Salen–Ti(III) (Table 4, entry 14) highlights that our system is less active (55% yield for **9**; 70% yield for [(Salen)Ti(III)Cl]), but it is more selective at low CO_2_ pressures. Contrasting with the highly selective formation of polycarbonate by compound **9**, the Salen–Ti complex produces a mixture of polycarbonate and cyclic carbonate (Table 4, entry 14). Surprisingly, when compound **9** is compared with [(Salen)Cr(III)Cl] (Table 4, entry 15), both catalytic systems are highly selective, but our Ti(III) catalyst is slightly more active, providing higher conversions.

Finally, the comparison of the catalytic activity of complex **9** with the most active Ti(IV) systems [(Boxdipy)TiCl]^21^ (0.05 mol %, 12 h, 20 bar, 45%, 13.0 kg/mol), [(Salalen)TiCl]^23^ (0.2 mol %, 10 h, 70 °C, 40 bar, 44%, 4.2 kg/mol), and [(ATP)^Me^TiO*i*Pr] (ATP = amino-tris(phenolate))^44^ (0.2 mol %, 4 h, 80 °C, 40 bar, 48%, 15.7 and 6.8 kg/mol) reveals that albeit our Ti(III) system is capable to mediate the copolymerization reaction at atmospheric pressure and relatively mild reaction temperatures, it exhibits lower catalytic activity (0.5 mol %, 18 h) and generates polycarbonate with moderate molecular weight (3.9 kg/mol).

## Conclusions

We describe the synthesis and characterization of bis(PDA)-Ti(III) species and their use for the functionalization of CO_2_ under atmospheric reaction conditions. Chemical reduction of the Ti(IV) precursors turned out to be the only productive route toward the low-valent titanium compound. Upon combination of X-ray studies, ^1^H NMR spectroscopy, reaction pressure monitoring, and DFT calculations, we disclose full details for the synthetic methodology from Ti(IV) to Ti(III). The reaction between two equivalents of the corresponding lithiated PDA ligand and the Ti(IV) chloride results in partial transmetalation, forming the heterobimetallic Ti(IV)/Li complexes. Then, the Ti(IV)/Li compounds react with [LiBHEt_3_] to generate first the Ti(IV)-bis(amido) compounds. These complexes are capable to accept a hydride fragment from [LiBHEt_3_], leading to a putative complex “[Li(thf)_4_][TiH(^Ar^PDA)_2_],” similar to the isolated species [Li(thf)_4_][Ti(NMe_2_)(^Ar^PDA)_2_] **7**. Finally, these titanium hydride species react through bimetallic reductive elimination to form the final Ti(III) compounds along with H_2_ release.

After an optimization process, the Ti(III) bis(diamido) **8** and **9** show good catalytic activity for the catalytic transformation of CO_2_ into polycarbonate via copolymerization with cyclohexene epoxide. Most relevant, the current studies provide a titanium species capable of operating under low CO_2_ pressures and selectively, so far only accessible for the bis-aryloxy N-heterocyclic carbene (NHC) titanium reported by Le Roux. Furthermore, the Ti(III) compounds display catalytic activity and selectivity similar to Salen–chromium compounds. Considering the structural versatility of the employed ligands and the levels of activity and selectivity in the copolymerization processes, the development of more efficient catalysts operating at lower catalyst loading with further epoxides, including biorenewable and those extracted as waste products, to generate polycarbonates of larger molecular weights is envisioned.

## Experimental Section

### General Considerations

All reactions were performed under a protective atmosphere using either standard Schlenk techniques (argon) or in an MBraun dry box (argon). [*d*_1_]-Chloroform and methanol were purchased from Sigma-Aldrich Chemicals and used as received. [*d*_6_]-Benzene and [*d*_8_]-tetrahydrofuran were purchased from Eurisotop and toluene, hexane, and tetrahydrofuran from Scharlab. Solvents were dried by heating to reflux over the appropriated drying agents: [*d*_6_]-Benzene, toluene, and hexane (Na/K alloy), [*d*_8_]-tetrahydrofuran (Na), and tetrahydrofuran (Na/Benzophenone) and distilled prior to use. CO_2_ (99.9993%) was commercially obtained from Linde Gas España and used without further purification. Commercially available reagents were purchased from Sigma-Aldrich Chemicals; [TiCl_4_(thf)_2_],^48^*N,N′-*bis(2,4,6-trimethylphenyl)-*o*-phenylenediamine (^Mes^PDAH_2_),^49^ [Li_2_(^Mes^PDA)(thf)_3_],^27b^*N,N*′-bis(2,6-isopropylphenyl)-*o-*phenylenediamine(^*i*Pr^PDAH_2_),^50^ [Li_2_(^*i*Pr^PDA)(thf)_3_],^27b^ [(Salen)Ti(III)Cl],^19^ and [(Salen)Cr(III)Cl]^47^ were synthesized as described in the literature. NMR spectra were recorded on a Varian Mercury-VX spectrometer operating at 300 MHz for ^1^H, 75 MHz for ^13^C{^1^H}, or on a Bruker Neo spectrometer operating at 400 MHz for ^1^H, 100 MHz for ^13^C{^1^H}, and 155.4 MHz for ^7^Li and on a Unity-500 Plus (500MHz for ^1^H) for variable temperature experiment. ^1^H, ^13^C{^1^H}, and ^7^Li chemical shifts are expressed in parts per million (δ, ppm) and referenced to residual solvent peaks. All coupling constants (*J*) are expressed in absolute values (Hz) and resonances are described as follows: s (singlet), d (doublet), hp (heptuplet), and m (multiplet). The NMR assignments were performed, in some cases, with the help of ^1^H,^13^C-HSQC and ^1^H,^13^C-HMBC experiments. Elemental analyses (C, H, N) were performed with a LECO CHNS-932 microanalyzer. Samples for IR spectroscopy were prepared as KBr pellets and recorded on the Bruker FT-IR-ALPHA II spectrophotometer (4000–400 cm^–1^). CW–EPR spectra were performed in a Bruker EMX spectrometer. Monitoring of H_2_ release was carried out in a Man on the Moon X102 kit micro-reactor in the glovebox. The molecular weights (*M*_n_) and the molecular mass distributions (*M*_w_/*M*_n_) of polymer samples were measured by gel permeation chromatography (GPC) performed on an Agilent 1260 Infinity II equipped with two GPC/columns PL gel 5 μm MIXED-D 300 × 7.5 mm and a G7162A refractive index detector. Calibration was performed with polystyrene (PS) standards in a range of molecular weights of 580–364,000 Da. MALDI-ToF-MS spectra were acquired with a Bruker Autoflex II ToF/ToF spectrometer (Billerica, MA, USA), using a nitrogen laser source (337 nm, 3 ns) in linear mode with a positive acceleration voltage of 20 kV.

### Synthesis of Complex [{TiCl(^*i*Pr^PDA)}(μ-^*i*Pr^PDA){Li(thf)}] (**3**)

A 100 mL Schlenk vessel was charged in the glovebox with [Li_2_(^*i*Pr^PDA)(thf)_3_] (**1**) (0.56 g, 0.88 mmol) and [TiCl_4_(thf)_2_] (0.147 g, 0.44 mmol) in 20 mL of hexane. The suspension was stirred for 1 h, filtered through a medium porosity glass frit to remove LiCl, and the resulting solution was dried under vacuum to yield **3** as a black solid (Yield: 75%, 0.35 g, 0.33 mmol). IR (KBr, cm^–1^): *ṽ* = 3057 (m), 2928 (m), 2867 (m) 1596 (s), 1527 (s), 1458 (s), 1319 (s), 1258 (m), 1170 (s), 1039 (s), 792 (m), 742 (m). ^**1**^**H NMR** (300 MHz, 298K, C_6_D_6_): 7.29–7.00 (m, 12H, C*H*_Ar_-^*i*^Pr), 6.85–6.72 (m, 4H, C_6_*H*_4_[N(*i*Pr)]_2_), 6.35–6.24 (m, 2H, C_6_*H*_4_[N(*i*Pr)]_2_), 5.40–5.30 (m, 2H, C_6_*H*_4_[N(*i*Pr)]_2_), 3.78–3.68 [m, 2H, C*H*(CH_3_)_2_], 3.66–3.55 (m, 2H, C*H*(CH_3_)_2_), 3.12–3.02 (m, 8H, thf), 3.00–2.80 (m, 4H, C*H*(CH_3_)_2_), 1.45 (d, 6H, *J* = 9 Hz, CH(C*H*_3_)_2_), 1.27 (d, 6H, *J* = 9 Hz, CH(C*H*_3_)_2_), 1.23 (d, 6H, *J* = 9 Hz, CH(C*H*_3_)_2_), 1.22 (d, 6H, *J* = 9 Hz, CH(C*H*_3_)_2_), 1.17 (d, 6H, *J* = 9 Hz, CH(C*H*_3_)_2_), 1.09 (d, 6H, *J* = 9 Hz, CH(C*H*_3_)_2_), 0.96 (d, 6H, *J* = 9 Hz, CH(C*H*_3_)_2_), 0.73 (d, 6H, *J* = 9 Hz, CH(C*H*_3_)_2_). ^**13**^**C-{**^**1**^**H}-NMR** (75 MHz, 298K, C_6_D_6_): δ 166.2, 148.0, 145.8,145.0, 142.7, 141.8, 139.4, 127.1 100.1 (*C*_q_), 127.1, 124.3, 124.2, 124.1, 123.9, 117.0, 116.5, 100.5 (*C*H_Ar_), 68.2 (*C*H_2_-thf), 28.2, 28.1, 27.9, 25.6, 25.5, 25.3, [*C*H(CH_3_)_2_, CH(*C*H_3_)_2_], 25.2 (*C*H_2_, thf), 25.1, 23.9, 23.0, 22.79 [*C*H(CH_3_)_2_, CH(*C*H_3_)_2_]. ^**7**^**Li NMR** (155.4 MHz, 298K, C_6_D_6_) δ 2.09. Elemental analysis (%) Calcd. for C_68_H_94_N_4_O_2_ClTiLi (MW = 1089.78): C, 74.95; H, 8.69; N, 5.14. Found: C, 74.78; H, 8.55; N, 4.98.

### Synthesis of Complex [{TiCl(^Mes^PDA)}(μ-^Mes^PDA){Li(thf)_2_}] (**4**)

A 100 mL Schlenk vessel was charged in the glovebox with [Li_2_(^Mes^PDA)(thf)_3_] (**2**) (0.52 g, 0.9 mmol) and [TiCl_4_(thf)_2_] (0.15 g, 0.45 mmol) in 20 mL of hexane. The suspension was stirred overnight. Then, the solvent was removed under reduced pressure, affording a purple solid, which was extracted with toluene and filtered through a medium porosity glass frit. Evaporation of toluene under vacuum yields **4** as a dark purple solid (Yield: 67%, 0.276 g, 0.301 mmol). IR (KBr, cm^–1^): *ṽ* = 3039 (m), 2916 (m), 2858 (m), 1597 (m), 1525 (s), 1446 (s), 1231 (s), 1014 (w), 885 (w), 742 (m), 560 (w). ^**1**^**H NMR** (300 MHz, 298K, C_6_D_6_) δ 7.07 (s, 2H, C*H*, C*H*_Ar_-Mes), 7.06–7.00 (m, 2H, C_6_*H*_4_[N(Mes)]_2_), 6.80 (s, 2H, C*H*, C*H*_Ar_-Mes), 6.73 (s, 2H, C*H*, C*H*_Ar_-Mes), 6.70 (s, 2H, C*H*, C*H*_Ar_-Mes), 6.63–6.56 (m, 2H, C_6_*H*_4_[N(Mes)]_2_), 6.41–6.35 (m, 2H, C_6_*H*_4_[N(Mes)]_2_), 5.44–5.38 (m, 2H, C_6_*H*_4_[N(Mes)]_2_), 3.38–3.26 (m, 8H, thf), 2.56 (s, 6H, C*H*_3_), 2.42 (s, 6H, C*H*_3_), 2.27 (s, 6H, C*H*_3_), 2.04 (s, 6H, C*H*_3_), 2.01 (s, 6H, C*H*_3_), 1.71 (s, 6H, C*H*_3_), 1.24–1.12 (m, 8H, thf). ^**13**^**C-{**^**1**^**H}-NMR** (75 MHz, 298K, C_6_D_6_) δ 162.4, 148.32, 146.9 (*C*_q_), 134.7, 129.5 (*C*H_Ar_), 129.5 (*C*_q_), 129.2, 129.1, 129.0, 127.0, 125.1, 117.9, 114.1, 101.6 (*C*H_Ar_), 68.0 (*C*H_2_, thf), 25.7 (*C*H_2_, thf), 21.2, 21.0, 20.3, 20.2, 19.3, 18.9 (*C*H_3_, Mes). ^**7**^**Li-RMN** (155.4 MHz, 298K, C_6_D_6_) δ 2.22. Elemental analysis (%) Calcd. for C_56_H_68_N_4_O_2_ClLiTi (MW = 919.44): C, 73.15; H, 7.45; N, 6.09. Found: C, 73.41; H, 7.63; N, 6.00.

### Synthesis of Complex [Ti(^*i*Pr^PDA)_2_] (**5**)

A 100 mL Carius tube fitted with a Young’s valve was charged in the glovebox with [{TiCl(^*i*Pr^PDA)}(μ-^*i*Pr^PDA){Li(thf)}] (**3**) (0.2 g, 0.18 mmol) and lithium dimethylamide (0.009 g, 0.18 mmol) in 10 mL of toluene. The reaction mixture was heated for 18 h at 40 °C. Then, the volatiles were removed under reduced pressure. The black solid was extracted with hexane, filtered through a medium porosity glass frit, and dried under vacuum to give rise to **5** as a black solid (Yield: 67%, 0.108 g, 0.12 mmol). IR (KBr, cm^–1^): *ṽ* = 3060 (w), 2963 (s), 2868 (m) 1597 (w), 1500 (m), 1459 (s), 1259 (m), 746 (m), 587 (w). ^**1**^**H NMR** (300 MHz, 298K, C_6_D_6_): δ 7.44–7.31 (m, 4H, C*H*_Ar_-^*i*^Pr), 7.27–7.17 (m, 3H, C*H*_Ar_-^*i*^Pr), 7.10–7.00 (m, 5H, C*H*_Ar_-^*i*^Pr), 6.97–6.86 (m, 2H, C_6_*H*_4_[N(*i*Pr)]_2_), 6.68–6.61 (m, 2H, C_6_*H*_4_[N(*i*Pr)]_2_), 6.61–6.50 (m, 2H, C_6_*H*_4_[N(*i*Pr)]_2_), 6.33–6.22 (m, 2H, C_6_*H*_4_[N(*i*Pr)]_2_), 2.96 (hp, 2H, *J* = 6 Hz, C*H*(CH_3_)_2_), 2.84 (hp, 2H *J* = 6 Hz, C*H*(CH_3_)_2_), 2.68 (hp, 2H, *J* = 6 Hz, C*H*(CH_3_)_2_), 2.03 (hp, 2H, *J* = 6 Hz, C*H*(CH_3_)_2_), 1.11 (d, 6H, *J* = 6 Hz, CH(C*H*_3_)_2_), 1.06 (d, 6H, *J* = 6 Hz, CH(C*H*_3_)_2_), 1.03 (d, 6H, *J* = 6 Hz, CH(C*H*_3_)_2_), 0.86 (d, 6H, *J* = 6 Hz, CH(C*H*_3_)_2_), 0.72 (d, 6H, *J* = 6 Hz, CH(C*H*_3_)_2_), 0.60 (d, 6H, *J* = 6 Hz, CH(C*H*_3_)_2_), 0.57 (d, 6H, *J* = 6 Hz, CH(C*H*_3_)_2_), 0.53 (d, 6H, *J* = 6 Hz, CH(C*H*_3_)_2_). ^**13**^**C-{**^**1**^**H}-NMR** (75 MHz, 298K, C_6_D_6_): 150.1, 144.5, 144.3, 143.7, 142.6, 142.4 (*C*_q_), 126.2, 125.6, 124.5, 124.2, 123.7, 123.4, 116.4 (*C*H_Ar_), 29.4, 28.8, 28.56, 28.50, 28.4, 27.9, 25.8, 25.7, 24.6, 24.4, 22.8, 22.7 (*C*H(CH_3_)_2_, CH(*C*H_3_)_2_). Elemental analysis (%) Calcd. for C_60_H_76_N_4_Ti (MW = 901.16): C, 79.97; H, 8.50; N, 6.22. Found: C, 79.77; H, 8.55; N, 6.48.

### Synthesis of Complex [Ti(^Mes^PDA)_2_] (**6**)

A toluene solution (20 mL) of [{TiCl(^Mes^PDA)}(μ-^Mes^PDA){Li(thf)_2_}] (**4**) (0.43 g, 0.47 mmol) was heated at 60 °C for 18 h. The reaction mixture was filtered through a medium porosity glass frit and dried under vacuum to produce compound **6** as a black solid (Yield: 85%, 0.290 g, 0.39 mmol). IR (KBr, cm^–1^): *ṽ* = 3046 (w), 2965 (w), 2915 (m), 2854 (w), 1597 (m), 1480 (s), 1258 (s), 1153 (m), 845 (m), 745 (m), 562 (w). ^**1**^**H NMR** (300 MHz, 298K, C_6_D_6_) δ 6.99 (m, 4H, C_6_*H*_4_[N(Mes)]_2_), 6.72 (s, 8H, C*H*_Ar_-Mes), 6.60 (m, 4H, C_6_*H*_4_[N(Mes)]_2_), 2.10 (s, 24H, C*H*_3_), 1.88 (s, 12H, C*H*_3_). ^**13**^**C-{**^**1**^**H}-NMR** (75 MHz, 298K, C_6_D_6_) δ 145.9, 134.7 (*C*_q_), 132.4, 129.5, 125.2, 117.3 (*C*H_Ar_), 21.00, 18.79 (*C*H_3_, Mes). Elemental analysis (%) Calcd. for C_48_H_52_N_4_Ti (MW = 732.84): C, 78.67; H, 7.15; N, 7.56. Found: C, 78.76; H, 7.07; N, 7.69.

### Synthesis of Complex [Li(thf)_4_][Ti(^Mes^PDA)_2_(N(CH_3_)_2_)] (**7**)

A 50 mL Schlenk vessel was charged in the glovebox with [{TiCl(^Mes^PDA)}(μ-^Mes^PDA){Li(thf)_2_}] (**4**) (0.1 g, 0.1 mmol) and lithium dimethylamide (0.005 g, 0.1 mmol) in 10 mL of benzene. The suspension was stirred for 3 days at 40 °C, and then the solvent was removed under reduced pressure. The product was extracted with toluene and filtered through a medium porosity glass frit. The filtrate was stored at room temperature affording species **7** as dark red crystals (Yield: 53%, 0.057 g, 0.053 mmol). IR (KBr, cm^–1^): *ṽ* = 2958 (m), 2915 (m), 2856 (m), 1599 (m), 1504 (s), 1483 (s), 1405 (m), 1257 (s), 1037 (m), 741 (m), 495 (w). ^**1**^**H NMR** (300 MHz, 298K, C_6_D_6_) δ 6.70 (s, 4H, C*H*_Ar_-Mes), 6.62 (s, 4H, C*H*_Ar_-Mes), 6.36–6.30 (m, 4H, C_6_*H*_4_[N(Mes)]_2_), 5.92–5.85 (m, 4H, C_6_*H*_4_[N(Mes)]_2_), 3.19 (s, 6H, N(C*H*_3_)_2_), 3.11 (m, 16H, thf), 2.38 (s, 12H, C*H*_3_), 2.36 (s, 12H, C*H*_3_), 2.10 (s, 12H, C*H*_3_), 1.21–1.14 (m, 16H, thf). ^**13**^**C-{**^**1**^**H}-NMR** (75 MHz, 298K, C_6_D_6_) δ 150.1 (*C*_q_), 134.5, 132.8 (*C*H_Ar_), 132.2 (*C*_q_), 129.2, 128.6, 128.5, 127.3, 125.6 (*C*H_Ar_), 43.9 (N(*C*H_3_)_2_), 21.3, 19.6, 19.4 (*C*H_3_, Mes). ^**7**^**Li NMR** (155.4 MHz, 298K, C_6_D_6_): δ 1.04. Elemental analysis (%) Calcd. for C_66_H_90_N_5_O_4_Ti (MW = 1072.28): C, 73.93; H, 8.46; N, 6.53. Found: C, 73.76; H, 8.27; N, 6.70.

### Synthesis of Complex [Li(thf)_4_][Ti(^*i*Pr^PDA)_2_] (**8**)

A 100 mL Carius tube fitted with a Young′s valve was charged in the glovebox with [{TiCl(^*i*Pr^PDA)}(μ-^*i*Pr^PDA){Li(thf)}] (**3**) (0.17 g, 0.16 mmol) and 15 mL of toluene. The toluene solution was cooled to 0 °C and then lithium triethylborohydride (1 M in thf, 0.16 mL, 0.16 mmol) was added. After stirring at room temperature for 18 h, the solution was filtered through a medium porosity glass frit. Then, the solvent was removed under reduced pressure. The solid was dissolved in pentane, and the resulting solution was cooled at −30 °C for 24 h, affording single dark crystals identified as **8** (Yield: 55%, 0.095 g, 0.09 mmol). Alternatively, complex **8** can be also prepared by reacting complex **5** (0.1 g, 0.11 mmol) with 1 eq of lithium triethylborohydride (1 M in thf, 0.11 mL, 0.11 mmol) during 18 h and room temperature. (Yield: 53%, 0.062 g, 0.053 mmol). IR (KBr, cm^–1^): *ṽ* = 3057 (w), 2964 (m), 2929 (s) 1459 (s), 1436 (s), 1248 (s), 1169 (m), 929 (m), 900 (w), 794 (w), 746 (w). Elemental analysis (%) Calcd. for C_76_H_108_N_4_O_4_LiTi (MW = 1196.53): C, 76.29; H, 9.10; N, 4.68. Found: C, 75.83; H, 8.94; N, 4.48.

### Synthesis of Complex [Li(thf)_4_][Ti(^Mes^PDA)_2_] (**9**)

A 50 mL Carius tube fitted with a Young’s valve was charged in the glovebox with the compound [{TiCl(^Mes^PDA)}(μ-^Mes^PDA){Li(thf)_2_}] (**4**) (0.56 g, 0.61 mmol) and 10 mL of toluene. To the toluene solution at 0 °C lithium triethylborohydride (1 M in thf, 0.61 mL, 0.61 mmol) was added. The reaction mixture was allowed to warm up to room temperature and stirred for 18 h. The resulting suspension was filtered through a medium porosity glass frit. The filtrate was concentrated to half volume under *vacuum* and cooled to −30 °C to afford **9** as dark green crystals. (Yield: 47%, 0.31 g, 0.3 mmol). Alternatively, complex **9** can be also obtained by reacting compound **6** (0.50 g, 0.68 mmol) with lithium triethylborohydride (1 M in thf, 0.68 mL, 0.68 mmol) during 18 h and room temperature. (Yield: 62%, 0.43 g, 0.42 mmol). IR (KBr, cm^–1^): *ṽ* = 2996 (m), 2915 (m), 1635 (m), 1542 (m), 1471 (s), 1258 (s), 1197 (m), 1149 (m), 1038 (m), 879 (s), 764 (m), 742 (m). Elemental analysis (%) Calcd. for C_64_H_84_N_4_O_4_TiLi (MW = 1028.21): C, 74.26; H, 8.23; N, 5.23. Found: C, 73.87; H, 8.20; N, 5.87.

### General Procedures for Catalytic Tests

All low-pressure reactions were carried out in a magnetically stirred Carius tube fitted with a Young’s valve.

A Carius tube fitted with a Young’s valve was charged in the glovebox with cyclohexene oxide (0.58–14.59 mmol), {[PPN]Cl} (0.017 g, 0.029 mmol), and titanium catalyst (0.014 mmol) with a magnetic stirrer bar. The argon atmosphere was replaced by 1 bar of CO_2_ using a Schlenk line and the reaction mixture was stirred for 18 h. The reaction crude was purified by evaporation of the excess of *cis*-CHO under reduced pressure. Then, polymers were dissolved in dichloromethane and precipitated with methanol to form a white solid. The isolated polymers were dried under vacuum at 50 °C for 48 h.

The conversion of cyclohexene oxide into poly(cyclohexene carbonate) (PCHC) was determined by normalization of the integrals of the methylene proton resonances in the ^1^H NMR spectra for the carbonate (δ = 4.65 ppm for PCHC and 4.00 ppm for *trans*-cyclic carbonate) and ether linkages (δ = 3.45 ppm) toward CHO (δ = 3.00 ppm) and expressed as a percentage of CHO conversion versus the theoretical maximum (100%).

The percentage of carbonate linkages was determined by ^1^H NMR spectroscopy of a sample and expressed as a percentage of carbonate linkages versus the theoretical maximum (100%), determined by comparison of the relative integrals of the resonances assigned to the carbonate (4.65 ppm for PCHC and 4.00 ppm for *trans-*cyclic carbonate) and ether (3.45 ppm) linkages, if present.

### Crystal Structure Determination of Complexes **3–9**

Single crystals for compounds **3**, **5**, and **8** were deposited from pentane solutions stored at −30 °C, while for complexes **4** and **6** crystals were grown up by slow diffusion of a toluene solution into a second layer of hexane. Compounds **7** and **9** were crystalized by slow evaporation of saturated benzene and tetrahydrofuran solutions, respectively.

The intensity data sets for **4**, **5**, **6**, and **9** were collected at 200 K on a Bruker-Nonius Kappa CCD diffractometer equipped with graphite-monochromated Mo Kα radiation (λ = 0.71073 Å) and an Oxford Cryostream 700 unit, while those for **3**, **7**, and **8** were collected at 150 K on a Bruker D8 Venture diffractometer equipped with multilayer optics for monochromatization and collimator, Mo Kα radiation (λ = 0.71073 Å), and an Oxford Cryostream 800 unit. Crystallographic data for all complexes are presented in Tables S4 and S5.

The structures were solved by applying intrinsic phasing (SHELXT)^51^ using the Olex2^52^ package and refined by least squares against F^2^ (SHELXL).^53^ All non-hydrogen atoms were anisotropically refined, while hydrogen atoms were placed at idealized positions and refined using a riding model.

### Computational Details

All DFT calculations have been carried out using the GAUSSIAN16 program.^54^ Geometry optimizations have been performed without any symmetry restrictions, taking into account dispersion effects with the Grimme and co-workers DFT-D3BJ correction^55,56^ at the B3LYP-D3BJ/def2SVP level of theory.^57−59^ After geometry optimization, analytical frequency calculations have been performed at the same level of theory to evaluate enthalpy and entropy corrections to the Gibbs energies at 298.15 K and to ensure that all frequencies were positive for all intermediates. Single-point calculations on the equilibrium geometries, including the effect of the solvent (toluene, via the self-consistent reaction field – SCRF – method using the SMD solvation model)^60^ and the dispersion effects (*E*_sp_), have been carried out at the B3LYP-D3BJ(SMD)/def2TZVP level of theory.^61^ Finally, the total Gibbs energy values (*G*) have been corrected using the GoodVibes code^62^ so that frequencies below 100 are not treated with the harmonic approximation, but rather with the quasi-harmonic approximation as described by Grimme.^63^

Effective oxidation states (EOS), spin-resolved effective fragment orbitals (EFOs), and fuzzy atom Mayer bond orders^64^ were obtained using APOST-3D^65^ using a 50 × 266 atomic grid for the numerical integrations and the topological fuzzy Voronoi cells (TFVC)^66^ for real-space partitioning.

## Data Availability

The optimized XYZ Cartesian coordinates at B3LYP-D3BJ/def2SVP level for all of the structures can be found in the following database link, in a very convenient format and allowing easy visualization and extraction of the XYZ file if needed: https://doi.org/10.19061/iochem-bd-4-52.

## Supporting Information Available

Effective oxidation state (EOS) analysis (Tables S1 and S2). Changes in Gibbs free energies and optimized geometries for the conversion of **3** to **5** and **4** to **6** (Table S3 and Figures S1–S4). EPR spectroscopy (Figure S5). Monitoring of H_2_ evolution over time (Figures S6 and S7). GPC, ^1^H NMR, and MALDI-ToF analysis of poly(cyclohexene carbonate) (Figures S8–S10). GC-MS of cyclohexene epoxide after stirring for 18 h at 50 °C under a CO_2_ atmosphere (Figure S11). Reaction between [^Ar^PDAH_2_] and [Ti(CH_2_Ph)_4_] (Figures S12 and S13). Reaction between [Li_2_(^Ar^PDA)(thf)_3_] and [TiCl_3_(thf)_3_] (Figures S14 and S15). Crystallographic data for compounds **3**–**9** (Tables S4 and S5). Van der Waals models for compounds **3** and **4** (Figure S16). Spectroscopical details for compounds **3**–**9** (Figures S17–S37) (DOCX)
