# Supplementary material for: Ti(III) Catalysts for CO2/Epoxide Copolymerization at Unusual Ambient Pressure Conditions
Source: Inorg Chem. 2023 Aug 31;62(37):14873–87. doi: 10.1021/acs.inorgchem.3c01249 (PMC10521022; doi:10.1021/acs.inorgchem.3c01249)
Supplement: Supplementary file 1 — ic3c01249_si_001.docx [file ic3c01249_si_001.docx]

Ti(III) Catalysts for CO_2_/epoxide Copolymerization at Unusual Ambient Pressure Conditions

Ignacio Sancho,^a^ Marta Navarro,^a^ Marc Montilla_,_^b^ Pedro Salvador,^b^ Cristina Santamaría,^a^ Josep M. Luis*^b^ and Alberto Hernán-Gómez*^a^

^a^ Departamento de Química Orgánica y Química Inorgánica, Instituto de Investigación Química “*Andrés M. del Río*” (IQAR), Universidad de Alcalá, Campus Universitario, E-28805 Alcalá de Henares, Madrid, Spain. ^b^ Institute of Computational Chemistry and Catalysis and Department of Chemistry, University of Girona, Campus de Montilivi, 17003 Girona, Catalonia, Spain.

Table of Contents

[1. Effective oxidation state (EOS) analysis 3](#_Toc137553221)

[2. Changes in Gibbs free Energy and optimized geometries for conversion of 3 to 5 and 4 to 6 4](#_Toc137553222)

[3. EPR Spectroscopy 8](#_Toc137553223)

[4. Monitoring of H_2_ evolution over time 8](#_Toc137553224)

[5. GPC and MALDI-ToF analysis of poly(cyclohexene carbonate) 10](#_Toc137553225)

[6. GC-MS of cyclohexene epoxide after stirring for 18h at 50 °C under a CO_2_ atmosphere (1 bar). 12](#_Toc137553226)

[7. Reaction between [^Ar^PDAH_2_] and [Ti(CH_2_Ph)_4_] 12](#_Toc137553227)

[8. Reaction between [Li_2_(^Ar^PDA)(thf)_3_] and [TiCl_3_(thf)_3_] 13](#_Toc137553228)

[9. Crystallographic data for compounds 3-9. 15](#_Toc137553229)

[10. Van der Waals models for compounds 3 and 4. 17](#_Toc137553230)

[11. Spectroscopical details for compounds 3-9. 18](#_Toc137553231)

[12. References 28](#_Toc137553232)

# Effective oxidation state (EOS) analysis

The concept of oxidation state (OS) is related to the electron distribution around the atoms, which can nowadays be described to an unprecedented precision with modern electronic structure methods. The effective oxidation state (EOS) analysis introduced by the group of Pedro Salvador is formally applicable to any molecular system and wavefunction on equal footing. The scheme uses Mayer’s effective fragment orbitals (EFOs)^1^ and their occupation numbers (λ), which are obtained independently for each atom/ligand and for each spin. These spin-resolved EFOs are sorted by decreasing occupation number, and individual electrons are assigned to the EFOs with the highest occupation number until the total number of electrons is reached. This procedure leads to an effective configuration of each ligand/atom, and hence its OS. The difference in the occupation between the last occupied (LO) and first unoccupied (FU) EFOs indicates to which extent the electron distribution can be pictured as a discrete ionic model. A simple reliability index, *R (%) = min (R_α_, R_β_)*, which quantifies to which extent the electronic structure can be described by the formal (integer) charge picture, can be derived from the frontier EFOs and introduced for each spin, σ, as:

| $R_{\sigma}\left( \% \right)=100\cdot\min\left( 1, \max\left( 0,\lambda_{LO}^{\sigma}-\lambda_{FU}^{\sigma}+1/2 \right) \right).$ | (1) |
| --- | --- |

The OS assignment is considered as undisputable when the difference in occupation of the frontier EFOs exceeds half an electron, leading to *R = 100*. When the two frontier EFOs from different fragments exhibit the same occupation number, two different equally plausible OS distributions are present with *R = 50*. This is the worst-case scenario in an OS assignation.

In order to obtain the EFOs, an underlying atomic partitioning scheme is necessary. The shape of the EFOs is rather insensitive to partitioning, while the occupation numbers may vary. The partial ionic character of the bonds is better captured by schemes such as QTAIM or Topological Fuzzy Voronoi Cells (TFVC).

**Tables and Figures**

The EOS results (which have a very clear assignation) are mentioned in the manuscript. The following tables show the effective oxidations states (EOS) and the EFO Occupations from which the Reliability Index of 100% is derived.

**Table S1**. For the optimized compound **3**, Effective Oxidation State (EOS), and EFO Occupations of each fragment.

|  |  | **EFO Occupations** | |
| --- | --- | --- | --- |
| **Fragment** | **Effective Oxidation State** | **Last Occupied** | **First Unoccupied** |
| 1 (Ti) | 4 | 0.964 | 0.222 |
| 2 (Cl) | -1 | 0.801 | 0.010 |
| 3 (PDA-N) | -2 | 0.765 | 0.040 |
| 4 (PDA-Ring) | -2 | 0.764 | 0.025 |
| 5 (Li) | 1 | 0.982 | 0.029 |

**Table S2**. For the optimized compound **4**, Effective Oxidation State (EOS) and EFO Occupations of each fragment.

|  |  | **EFO Occupations** | |
| --- | --- | --- | --- |
| **Fragment** | **Effective Oxidation State** | **Last Occupied** | **First Unoccupied** |
| 1 (Ti) | 4 | 0.964 | 0.220 |
| 2 (Cl) | -1 | 0.805 | 0.009 |
| 3 (PDA-N) | -2 | 0.763 | 0.027 |
| 4 (PDA-Ring) | -2 | 0.771 | 0.025 |
| 5 (Li) | 1 | 0.982 | 0.030 |

# Changes in Gibbs free Energy and optimized geometries for conversion of 3 to 5 and 4 to 6

The transformation of **4** to **6** (and **3** to **5**) considering a single Lithium cation or two of them, is discussed in the manuscript. The corresponding change in Gibbs free energy and geometries and are shown here:

**Table S3**. Change in reaction Gibbs free energy (ΔG, in kcal/mol) for the formation of 5 and 6 from compounds 3 and 4, respectively, including one or two Li^+^ in the reactant complex.

|  | ΔG° |
| --- | --- |
| Ar = Mes \| 1 Li^+^ (4 → 6) | -2.08 |
| Ar = Mes \| 2 Li^+^ (4 → 6) | -17.21 |
| Ar = *i*Pr \| 1 Li^+^ (3 → 5) | 12.76 |
| Ar = *i*Pr \| 2 Li^+^ (3 → 5) | 0.72 |

| 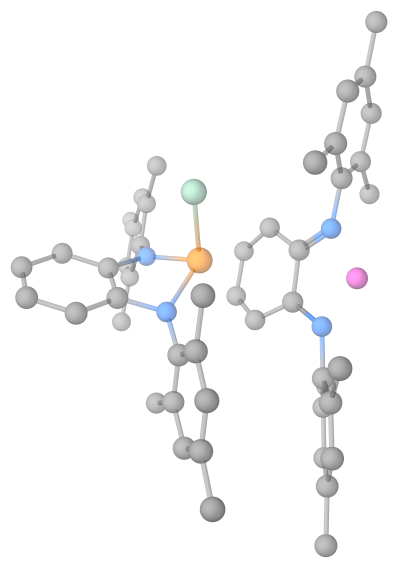 | 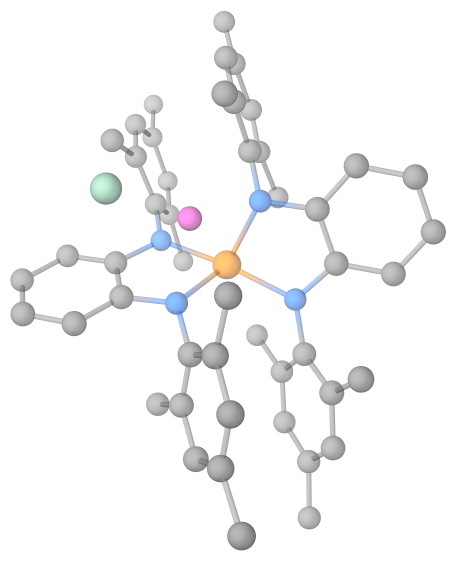 |
| --- | --- |

**Figure S1.** B3LYP‑D3BJ/def2SVP optimized geometries for **4** (left) and **6** (right), considering a single Lithium cation (ΔG=‑2.08 kcal/mol). The hydrogens are hidden for clarity, and the following colour code is used for the atoms: blue for nitrogen, grey for carbon, green for chlorine, purple for lithium, and orange for titanium.

| 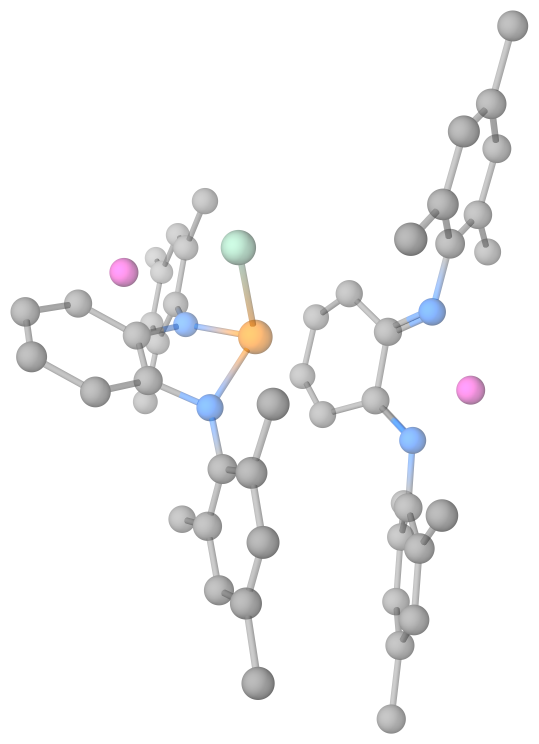 | 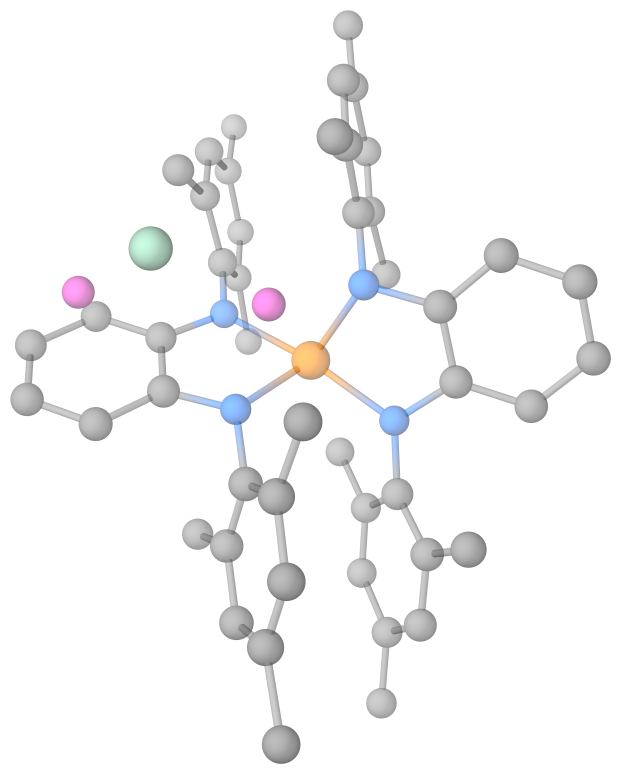 |
| --- | --- |

**Figure S2.** B3LYP‑D3BJ/def2SVP optimized geometries for **4** (left) and **6** (right), considering two Lithium cations (ΔG=‑17.21 kcal/mol). The hydrogens are hidden for clarity, and the following colour code is used for the atoms: blue for nitrogen, grey for carbon, green for chlorine, purple for lithium, and orange for titanium.

| 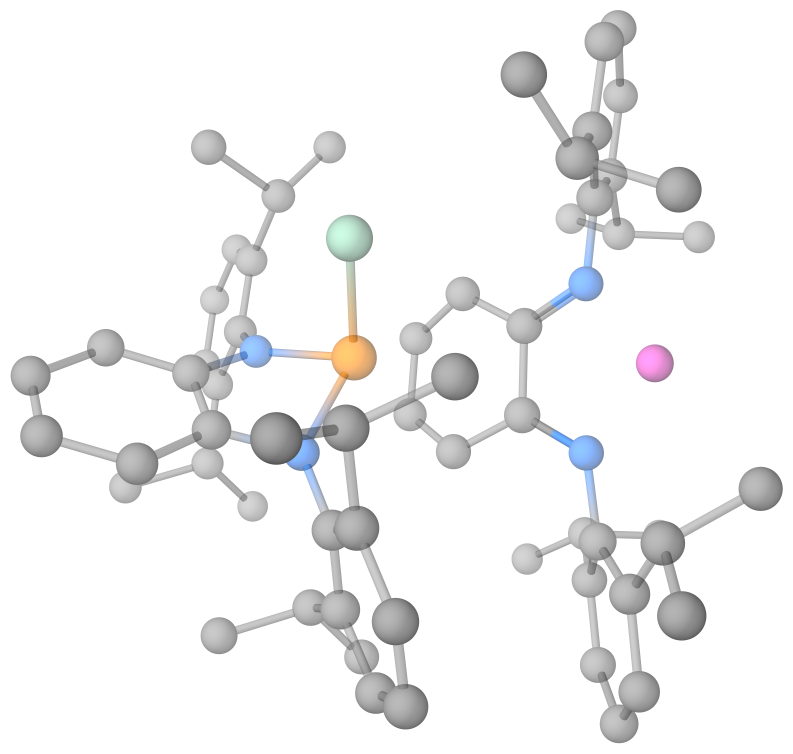 | 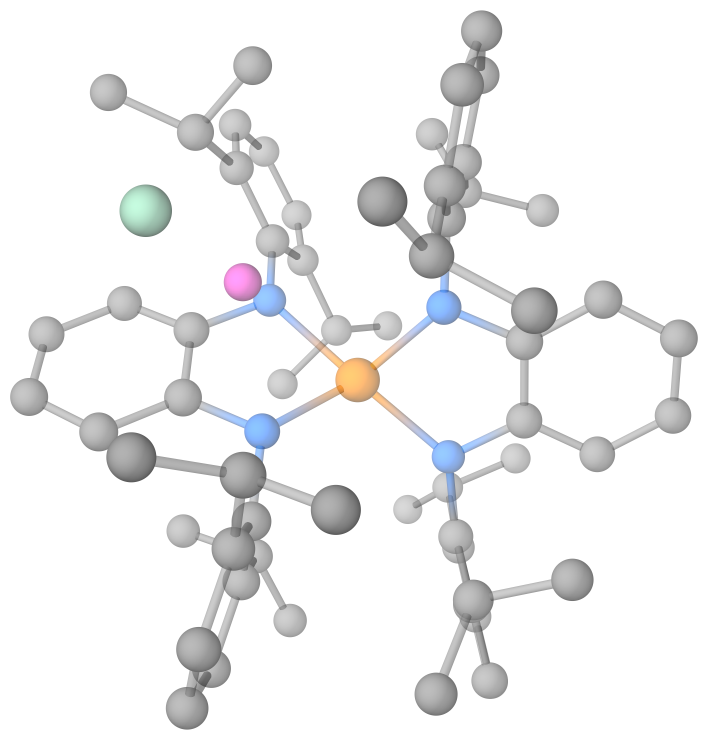 |
| --- | --- |

**Figure S3.** B3LYP‑D3BJ/def2SVP optimized geometries for **3** (left) and **5** (right), considering a single Lithium cation (ΔG=12.76 kcal/mol). The hydrogens are hidden for clarity, and the following colour code is used for the atoms: blue for nitrogen, grey for carbon, green for chlorine, purple for lithium, and orange for titanium.

| 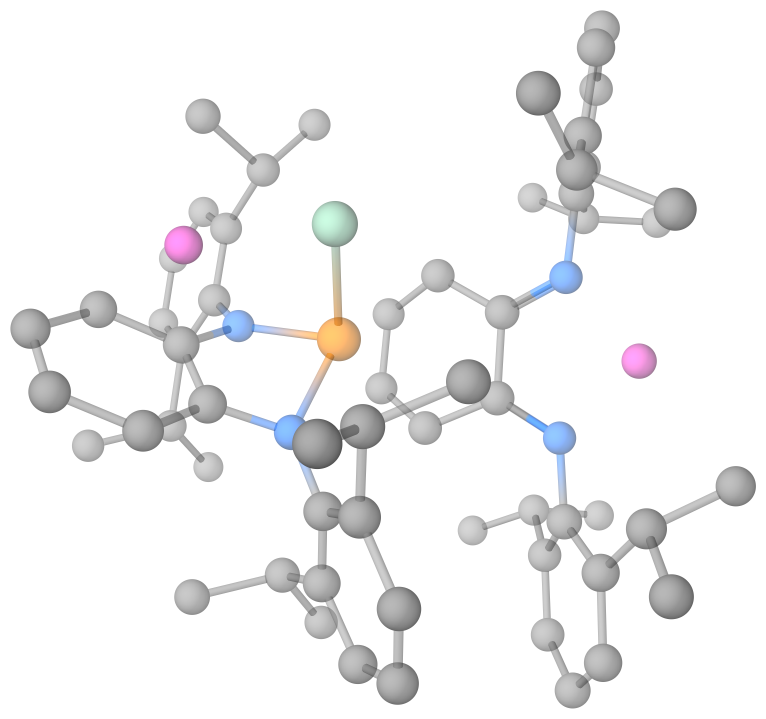 | 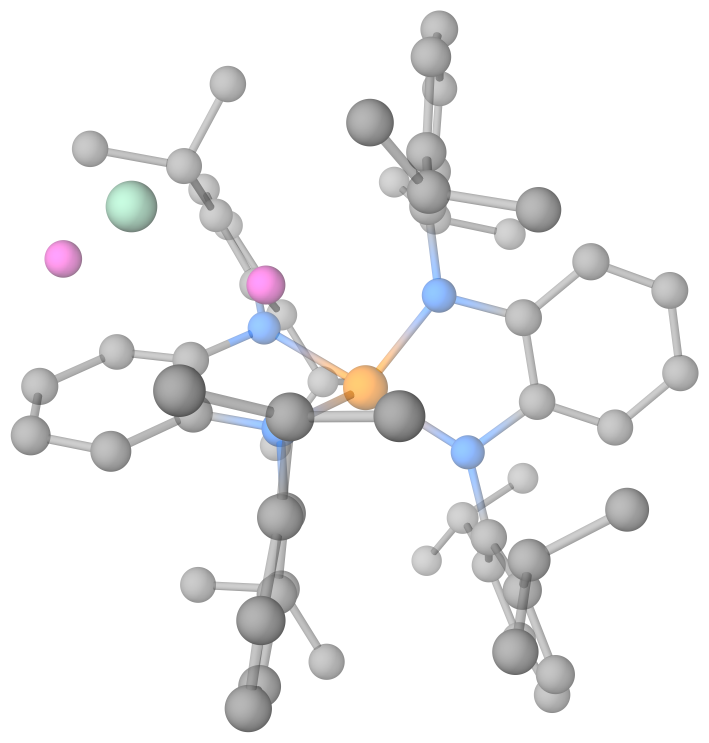 |
| --- | --- |

**Figure S4.** B3LYP‑D3BJ/def2SVP optimized geometries for **3** (left) and **5** (right), considering two Lithium cations (ΔG=0.72 kcal/mol). The hydrogens are hidden for clarity, and the following colour code is used for the atoms: blue for nitrogen, grey for carbon, green for chlorine, purple for lithium, and orange for titanium.

**DFT XYZ coordinates of geometry optimized structures**

The optimized XYZ Cartesian coordinates at B3LYP‑D3BJ/def2SVP level for all the structures can be found in the following database link, in a very convenient format and allowing easy visualization and extraction of the XYZ file if needed: <https://doi.org/10.19061/iochem-bd-4-52>

# EPR Spectroscopy

A 3 mM frozen thf solution of compounds **8** and **9** was registered in a Bruker EMX spectrometer.

**Experimental parameters**:

Temperature = 150 K; MW power = 0.2 mW; Receiver gain = 2.00 × 10^3^; modulation frequency = 100 kHz; modulation amplitude = 4.0 G.

**Simulation parameters** (performed using the Easyspin toolbox in Matlab):^2^

Compound **8**: g = [1.978, 1.949]; lwpp = [1.4413 0.7000]

Compound **9**: g = [1.972, 1.935]; lwpp = [0.4818, 0.3612]; gStrain = [0.0087 0.0098]

| 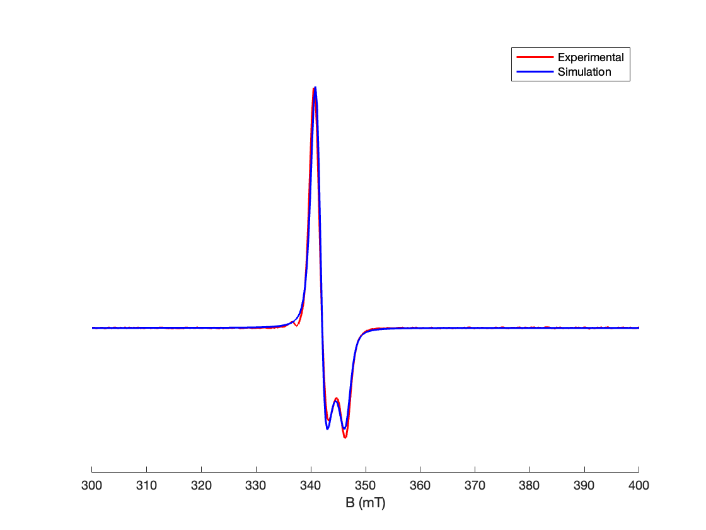 | 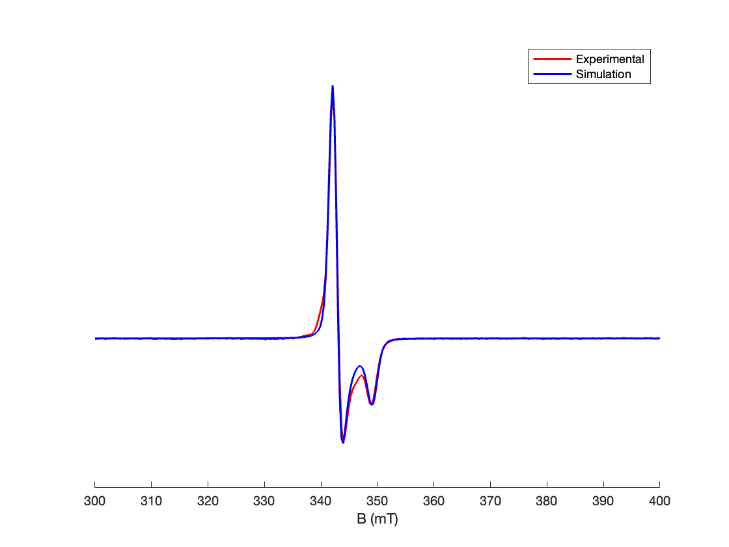 |
| --- | --- |

Figure S5. Low-temperature (77 K) EPR spectra of 8 (left, g_⊥_ = 1.978 and g_||_ = 1.950) and 9 (right, g_⊥_ = 1.972 and g_||_ = 1.935)

# Monitoring of H_2_ evolution over time

 Eq. 1

The hydrogen production during the reaction of complex **6** with [LiBEt_3_H] was monitored through the Man on the Moon X102 Kit (<https://www.manonthemoontech.com/index.html>), which permits monitoring the variation of pressure inside a closed glass reactor, using an electronic pressure transducer.

The reaction flask is connected to a switchable 3-way valve via a Thorion screw through polyamide tubing. The valve can be switched between two positions, one of them connecting the reactor vessel to the exterior. The other position connects the flask to the pressure transducer.

A round-bottom 20 mL flask was charged in the glovebox with complex **6** (0.13 mmol, 0,1 g) and 5 mL of tetrahydrofuran. Then, the flask was connected to the switchable three-way valve through a Torion screw. While stirring the reaction mixture at room temperature (298 K), the valve was switched to the pressure transducer which is connected via wireless to the software. Once the pressure measurement was stabilized, lithium triethylborohydride (1 M in thf, 0.2 mL, 0.2 mmol) was added. Then kinetic profile was recorded until constant pressure in the micro-reactor was reached (10 h). The number of H_2_ equivalents released was calculated assuming ideality for hydrogen gas and applying the PV = nRT.

The calculated number of H_2_ mmol is 0.08 which is consistent with the release of 0.5 equivalents (0.1 mmol) of H_2_ in the transformation of 0.2 mmol of compound **6** to **9**, according to equation 1.


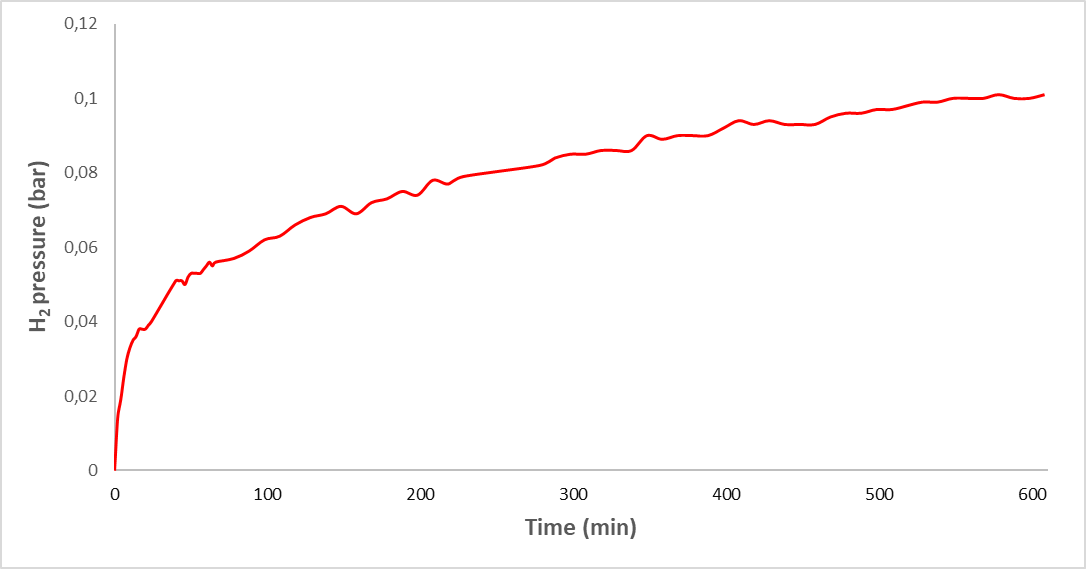


**Figure S6.** Pressure Monitoring for the chemical reduction of compound **6**.


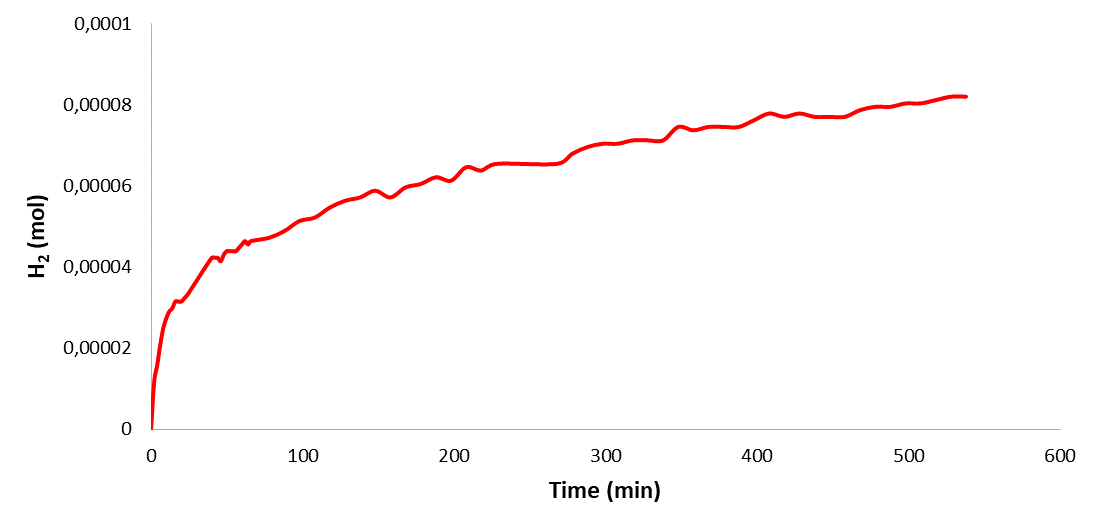


**Figure S7.** Pressure Monitoring for the chemical reduction of compound **6**.

# GPC, ^1^H NMR and MALDI-ToF analysis of poly(cyclohexene carbonate)


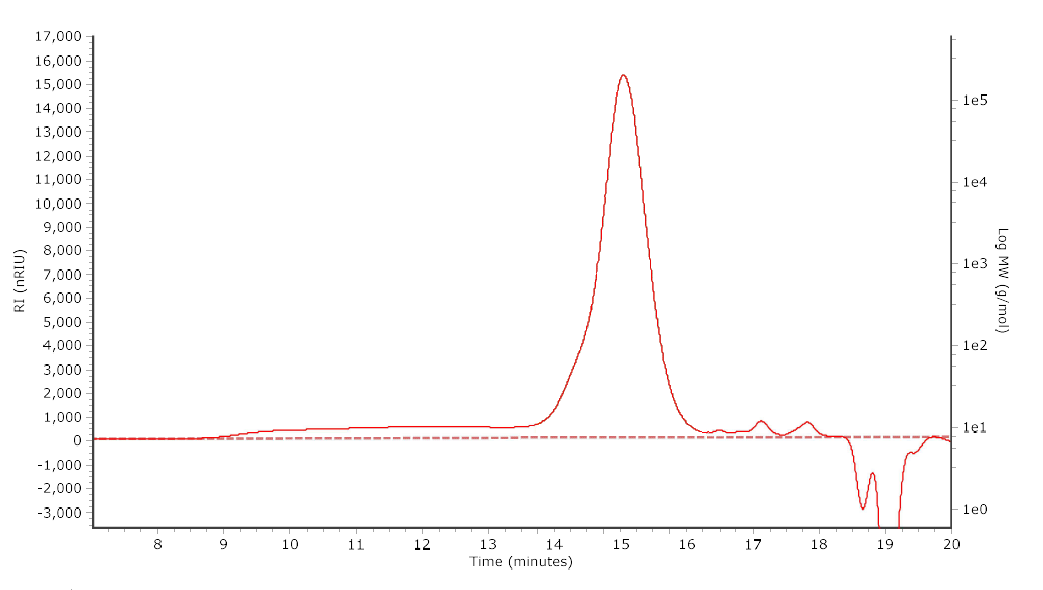


**Figure S8**. GPC trace of poly(cyclohexene carbonate) obtained by complex **9** at 50 ⁰C and 1 bar CO_2_ (Table 4, entry 9).

MALDI-ToF spectrum displays three series of peaks differing in the ending-groups. Within each series, peaks are separated by a molecular mass of 142 Da which corresponds to one cyclohexane carbonate unit (C_7_H_10_O_3_) (Figure S9). The spectrum shows one mayor series with a chlorine and hydroxyl as chain end groups (). The second and third population can be assigned to the following molecular formula [(C_7_H_10_O_3_)_n_+H+(OC_3_H_7_)]+Na^+^ () and [(C_7_H_10_O_3_)_n_+H+(OC_6_H_11_)]+Na^+^ () with alkoxide and hydroxyl ending groups. The alkoxide fragments are most likely formed upon minor side and stoichiometric reactions of the titanium catalyst with the epoxide, previous to the copolymerization process, as it has been reported for similar metal mediated copolymerization processes.^3^ Although hydrolysis processes can not be fully ruled out, GC-MS and ^1^H NMR analysis of cyclohexene epoxide after being expose to CO_2_ under the reaction conditions employed during catalysis (18h, 50 °C) did not show the presence of any organic alcohol (Figure S11).

**Figure S9.** MALDI-ToF mass spectrum of poly(cyclohexenecarbonate) using catalyst **9** at 50 °C and 1 bar CO_2_ (Table 4, entry 9).

**Figure S10**. ^1^H-NMR spectrum (300MHz, 298K, CDCl_3_) of poly(cyclohexenecarbonate) using catalyst **9** at 50 °C and 1 bar CO_2_ (Table 4, entry 9).

# GC-MS of cyclohexene epoxide after stirring for 18h at 50 °C under a CO_2_ atmosphere (1 bar).

**Figure S11**. GC-MS of cyclohexene epoxide after stirring for 18h at 50 °C under a CO_2_ atmosphere (1 bar).

# Reaction between [^Ar^PDAH_2_] and [Ti(CH_2_Ph)_4_]


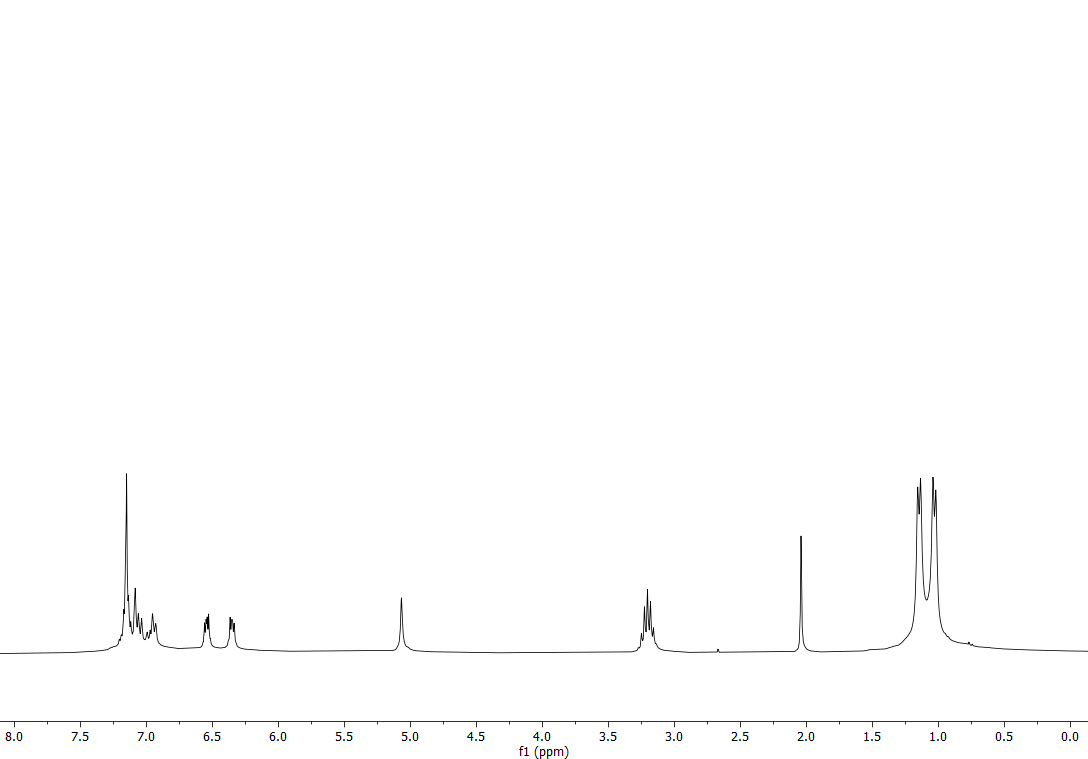


N*H*(Ar)

[Ti(C*H*_2_Ph)_4_]

**Figure S12.** ^1^H-NMR spectrum (300MHz, 298K, C_6_D_6_) for reaction between (^iPr^PDAH_2_) (0.15 g, 0.234 mmol) and [Ti(CH_2_Ph)_4_].(0.043 g, 0.117 mmol) at 110ºC, 18 h.


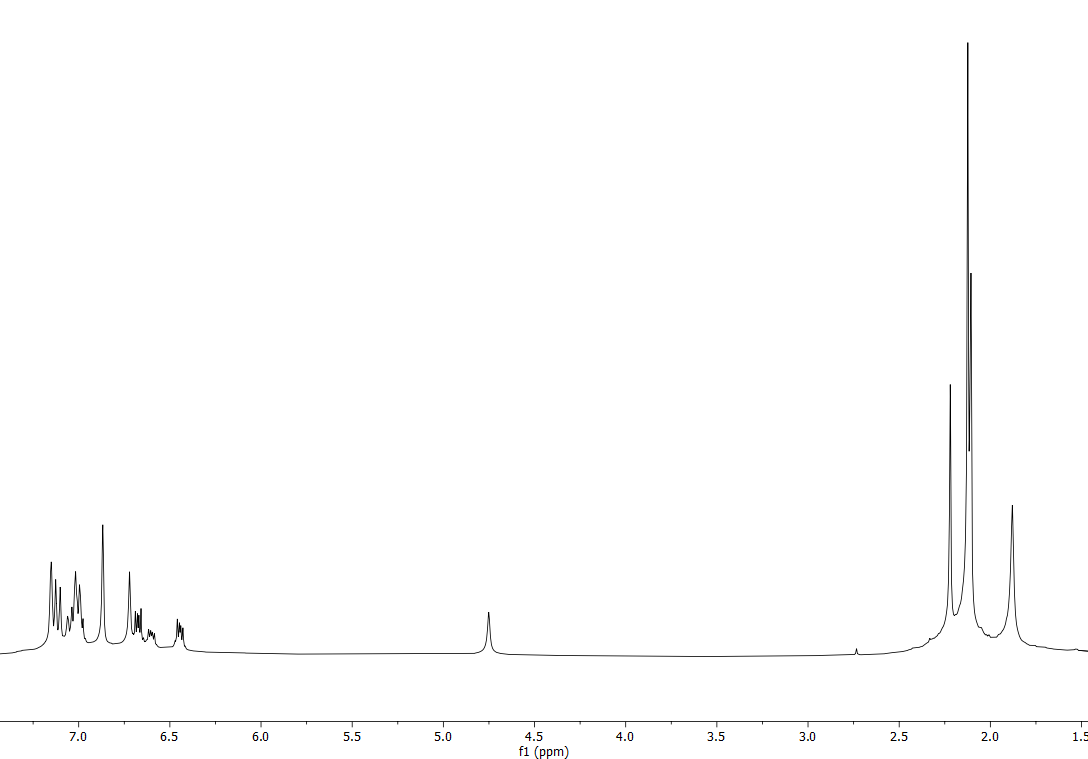


[Ti(^Mes^PDA)_2_]

N*H*(Ar)

**Figure S13**. ^1^H-NMR spectrum (300MHz, 298K, C_6_D_6_) for reaction between (^Mes^PDA) (0.1g, 0.145mmol) and [Ti(CH_2_Ph)_4_].(0.060 g, 0.290 mmol) at 110ºC, 18h.

# Reaction between [Li_2_(^Ar^PDA)(thf)_3_] and [TiCl_3_(thf)_3_]


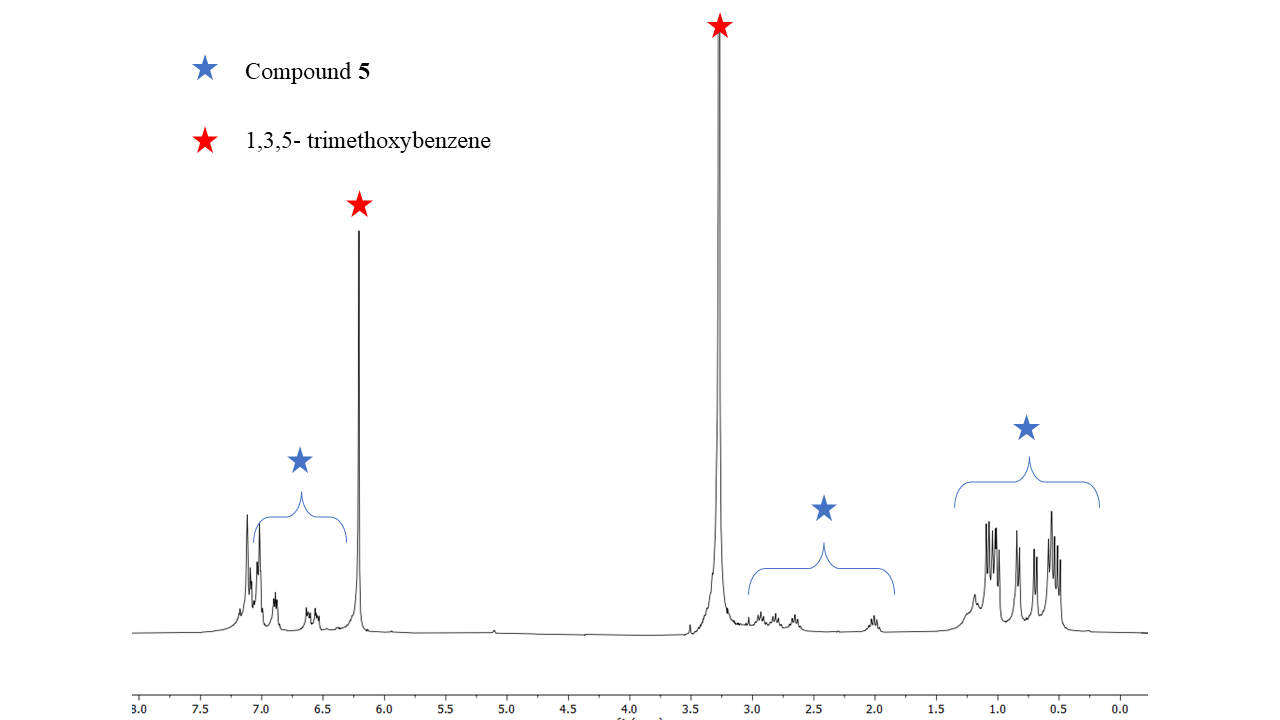


**Figure S14**. ^1^H-NMR spectrum (300MHz, 298K, C_6_D_6_) of the isolated solid from the reaction between [Li_2_(^iPr^PDA)(thf)_3_] and [TiCl_3_(thf)_3_] using 1,3,5-trimethoxybenzene as standard.


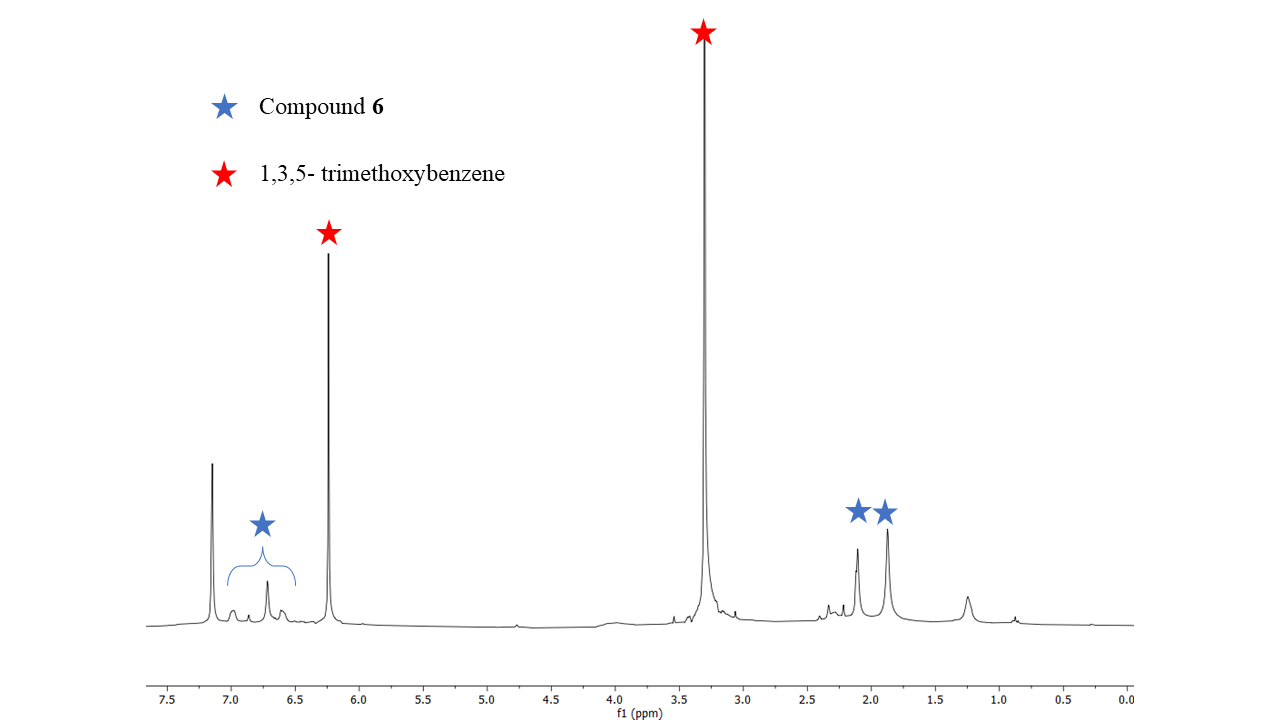


**Figure S15**. ^1^H-NMR spectrum (300MHz, 298K, C_6_D_6_) of the isolated solid from the reaction between [Li_2_(^Mes^PDA)(thf)_3_] and [TiCl_3_(thf)_3_] using 1,3,5-trimethoxybenzene as standard.

# Crystallographic data for compounds 3-9.

**Table S4**. Selected crystallographic and refinement parameters.

|  | **3** | **4** | **5** | **6** |
| --- | --- | --- | --- | --- |
| Empirical formula | C64H84ClLiN4OTi | C60H76ClLiN4O3Ti | C60H76N4Ti | C54H65N4Ti |
| Molecular Weight | 1015.64 | 991.53 | 901.14 | 818.00 |
| Temperature (K) | 150 | 200 | 200 | 200 |
| Wavelength (Å) | 0.71073 | 0.71073 | 0.71073 | 0.71073 |
| Crystal System | monoclinic | triclinic | Triclinic | triclinic |
| Space Group | P2_1_/c | P-1 | P-1 | P-1 |
| a (Å) | 12.8123(4) | 10.9765(11) | 11.6630(3) | 11.7378(8) |
| b (Å) | 23.0262(7) | 14.1066(16) | 12.9658(4) | 14.7654(11) |
| c (Å) | 20.3246(6) | 20.0516(18) | 19.8978(7) | 15.7113(12) |
| a (º) | 90 | 74.838(8) | 72.388(2) | 66.850(5) |
| b (º) | 96.5630(10) | 85.887(9) | 82.609(2) | 73.640(6) |
| c (º) | 90 | 87.224(7) | 74.745(2) | 70.584(5) |
| Cell Volume (Å ^3^) | 5956.8(3) | 2987.6(5) | 2762.92(15) | 2325.3(3) |
| Z | 4 | 2 | 2 | 2 |
| *ρ* Calc (g·cm^-3^) | 1.132 | 1.102 | 1.083 | 1.168 |
| μ (mm^-1^) | 0.231 | 0.231 | 0.194 | 0.224 |
| *F* (000) | 2184 | 1060 | 972 | 878 |
| 2θ max (º) | 55.02 | 51 | 53.44 | 51 |
| Index Ranges | -16≤*h*≤16  -29≤*k*≤29  -26≤*h*≤26 | -13≤*h*≤13  -17≤*k*≤17  -24≤*h*≤24 | -14≤*h*≤14  -16≤*k*≤16  -25≤*h*≤25 | -14≤*h*≤14  -17≤*k*≤17  -19≤*h*≤19 |
| Reflections collected | 109353 | 169560 | 176927 | 38103 |
| Reflections Unique | 13641 | 11100 | 11635 | 8606 |
| Reflections obs. | 9684 | 8132 | 9331 | 4520 |
| *R*_int_ | 0.0798 | 0.1064 | 0.0589 | 0.1283 |
| No. Parameters | 665 | 643 | 614 | 537 |
| Goodnes of Fit on *F*^2^ (GOF) | 1.037 | 1.174 | 1.056 | 1.017 |
| Final *R* indices [*I*>2s(*I*)] | 0.0451 | 0.0724 | 0.0566 | 0.0663 |
| *R* indices (all data) | 0.1153 | 0.1511 | 0.1903 | 0.1868 |
| Largest diff. peak and hole (e Å^-3^) | 0.362, -0.378 | 0.408, -0.361 | 0.740, -0.310 | 0.545, -0.382 |

**Table S5**. Selected crystallographic and refinement parameters.

|  | **7** | **8** | **9** |
| --- | --- | --- | --- |
| Empirical formula | C67H83LiN5O2Ti | C76H108LiN4O4Ti | C128H168Li2N8O8Ti2 |
| Molecular Weight | 1045.22 | 1196.50 | 2056.37 |
| Temperature (K) | 150 | 150 | 200 |
| Wavelength (Å) | 0.71073 | 0.71073 | 0.71073 |
| Crystal System | triclinic | monoclicnic | orthorombic |
| Space Group | P-1 | P2_1_/C | Pbca |
| a (Å) | 14.0649(16) | 12.7625(5) | 22.6288(6) |
| b (Å) | 15.1907(18) | 17.3696(8) | 21.7322(6) |
| c (Å) | 16.359(2) | 35.7913(16) | 47.1881(15) |
| a (º) | 70.222(4) | 90 | 90 |
| b (º) | 79.213(4) | 97.587(2) | 90 |
| c (º) | 68.983(4) | 90 | 90 |
| Cell Volume (Å ^3^) | 3061.5(6) | 7864.7(6) | 23205.9(1) |
| Z | 2 | 4 | 8 |
| *ρ* Calc (g·cm^-3^) | 1.134 | 1.011 | 1.177 |
| μ (mm^-1^) | 0.186 | 0.153 | 0.197 |
| *F* (000) | 1122 | 2596 | 8848 |
| 2θ max (º) | 51.36 | 51.44 | 55.84 |
| Index Ranges | -17≤*h*≤17  -18≤*k*≤12  -19≤*h*≤19 | -15≤*h*≤15  -21≤*k*≤21  -43≤*h*≤43 | -29≤*h*≤29  -28≤*k*≤28  -62≤*h*≤62 |
| Reflections collected | 52791 | 107394 | 285858 |
| Reflections Unique | 11572 | 14948 | 27690 |
| Reflections obs. | 8089 | 9830 | 21207 |
| *R*_int_ | 0.0913 | 0.0719 | 0.0688 |
| No. Parameters | 731 | 855 | 1399 |
| Goodnes of Fit on *F*^2^ (GOF) | 1.063 | 1.041 | 1.064 |
| Final *R* indices [*I*>2s(*I*)] | 0.0792 | 0.0717 | 0.0643 |
| *R* indices (all data) | 0.2523 | 0.2201 | 0.1665 |
| Largest diff. peak and hole (e Å^-3^) | 0.764, -0.822 | 0.471, -0.299 | 1.530, -0.687 |

# Van der Waals models for compounds 3 and 4.

| 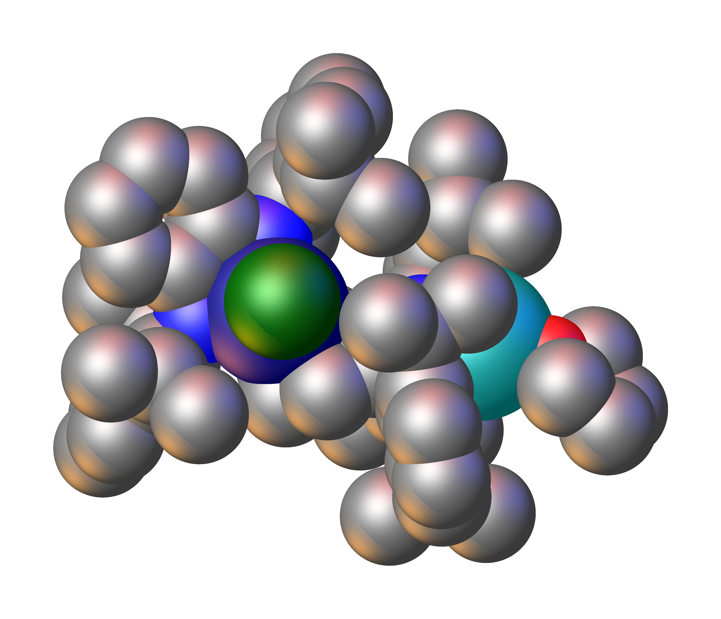 | 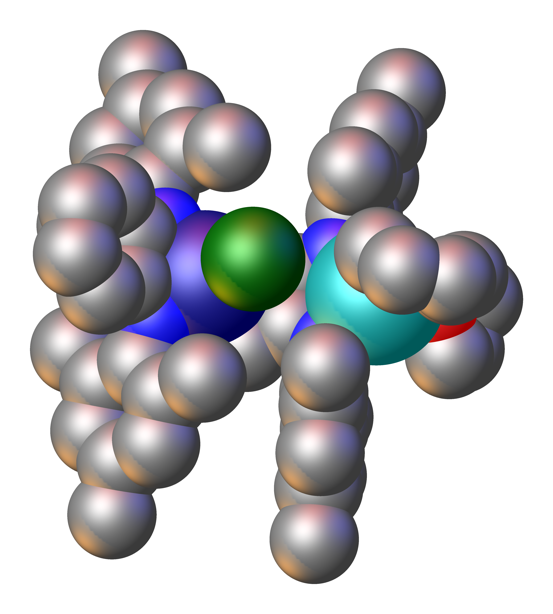 |
| --- | --- |

**Figure S16**. Solid state structure of compounds **3** (left) and **4** (right) using van der Waals model.

# Spectroscopical details for compounds 3-9.


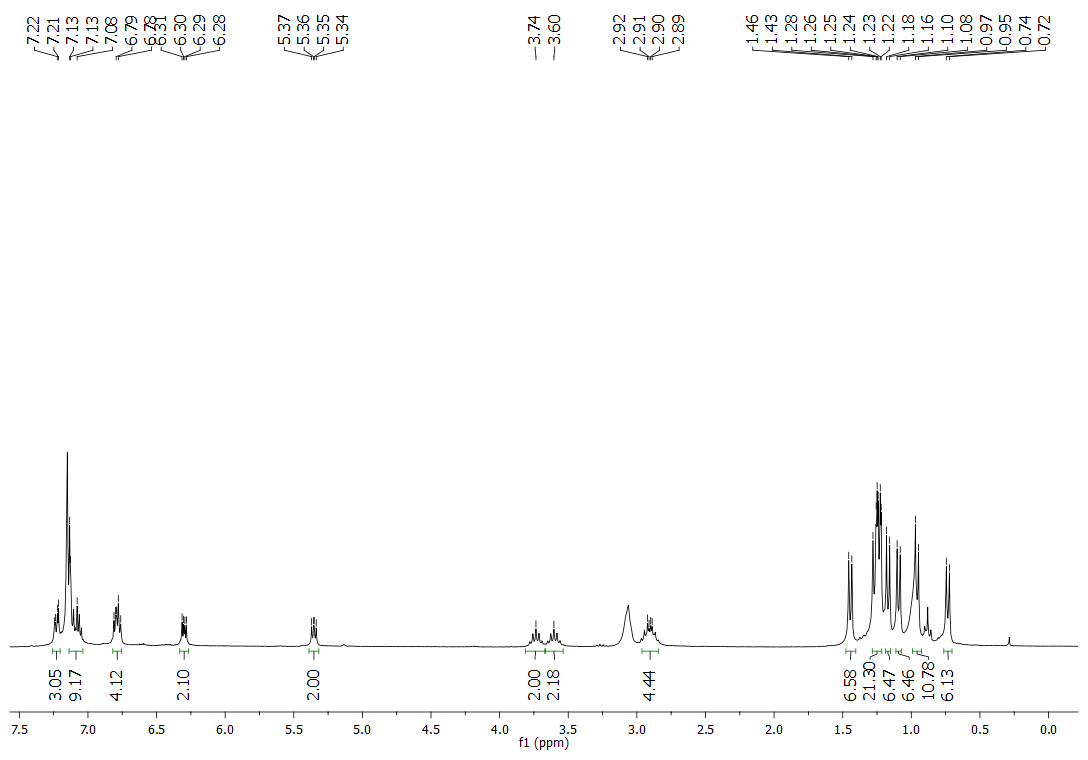
**Figure S17.** ^1^H-NMR spectrum (300MHz, 298K, C_6_D_6_) for complex [{TiCl(*^i^*^Pr^PDA)}(μ-*^i^*^Pr^PDA){Li(thf)}] (**3**).


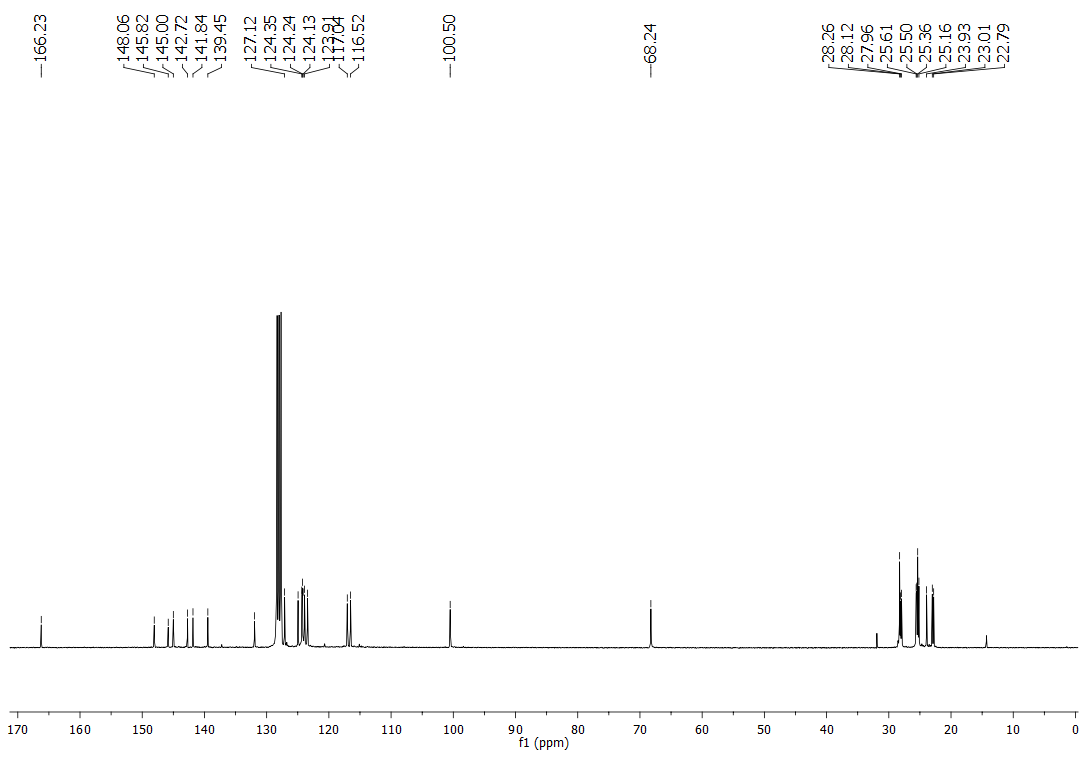


**Figure S18.** ^13^C-{^1^H}-NMR spectrum (75MHz, 298K, C_6_D_6_) for complex [{TiCl(^iPr^PDA)}(μ-^iPr^PDA){Li(thf)}](**3**).


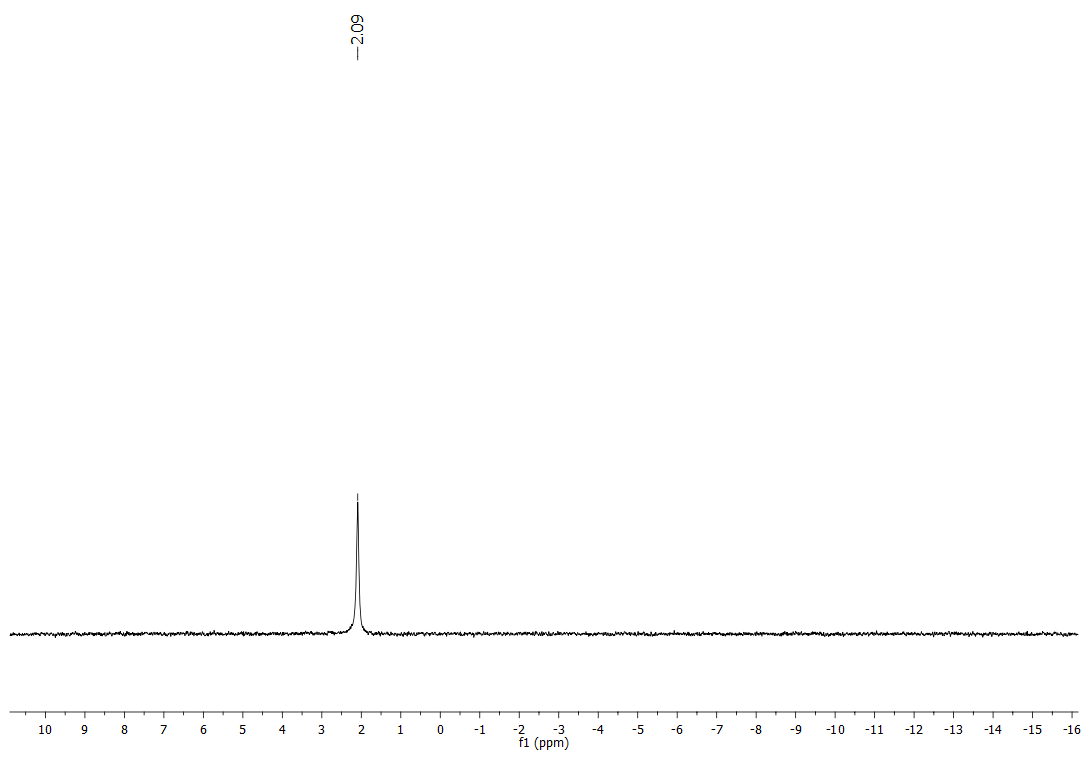


**Figure S19.** ^7^Li-NMR spectrum (155.4MHz, 298K, C_6_D_6_) for complex [{TiCl(^iPr^PDA)}(μ-^iPr^PDA){Li(thf)}](**3**).


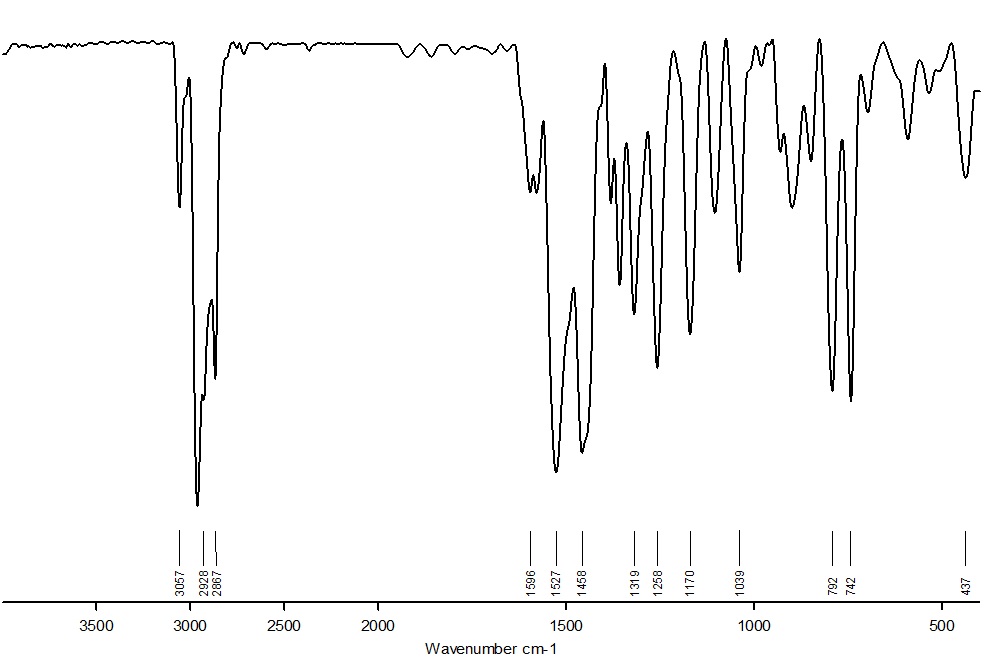


**Figure S20.** FTIR spectrum of complex [{TiCl(*^i^*^Pr^PDA)}(μ-*^i^*^Pr^PDA){Li(thf)}](**3**)


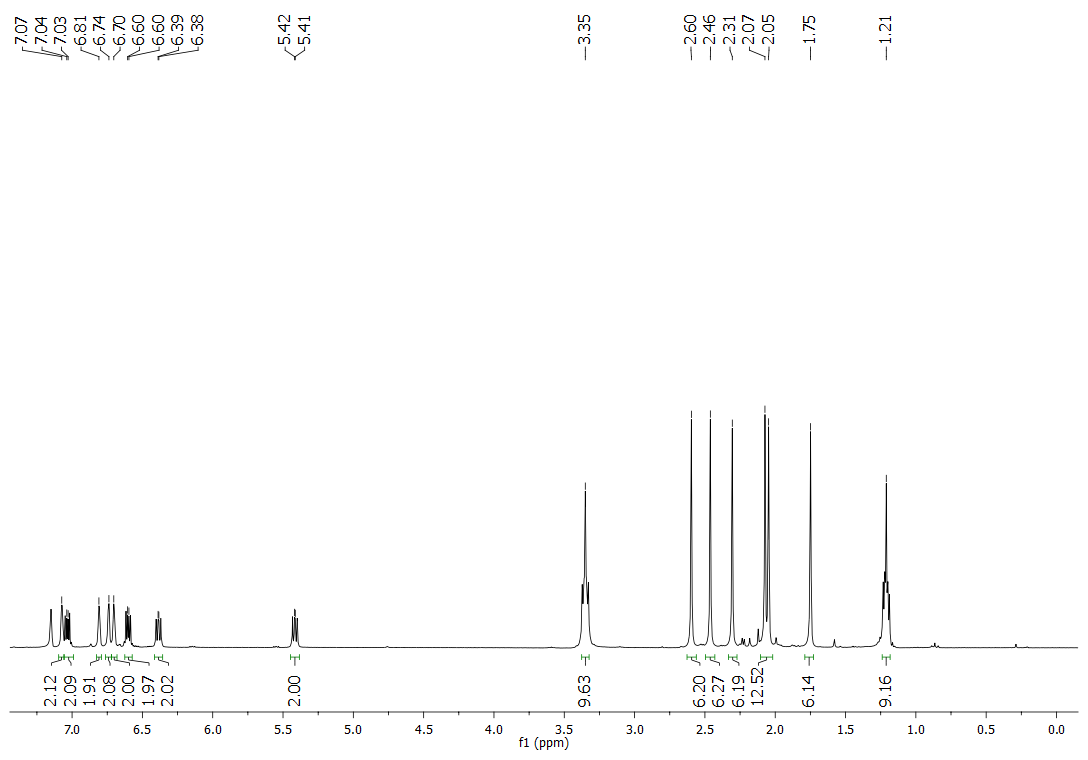
**Figure S21.** ^1^H-NMR spectrum (300MHz, 298K, C_6_D_6_) for complex [{TiCl(^Mes^PDA)}(μ-^Mes^PDA){Li(thf)_2_}] (**4**).


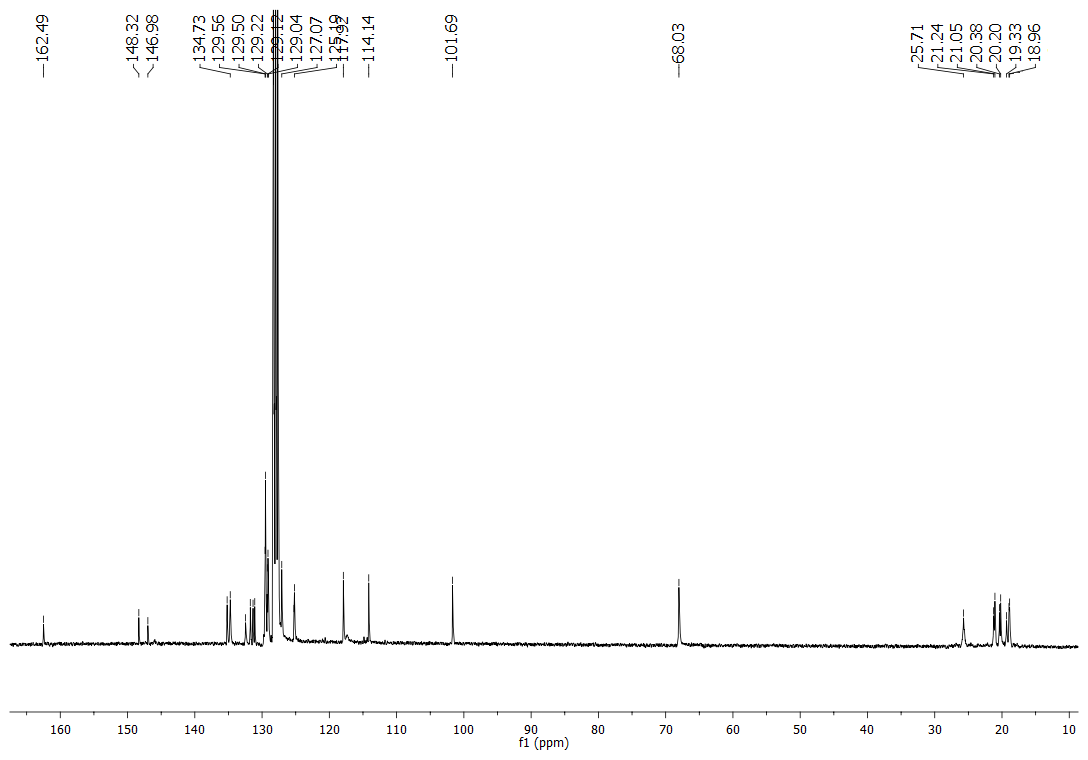


**Figure S22.** ^13^C-{^1^H}-NMR spectrum (75MHz, 298K, C_6_D_6_) for complex [{TiCl(^Mes^PDA)}(μ-^Mes^PDA){Li(thf)_2_}] (**4**).


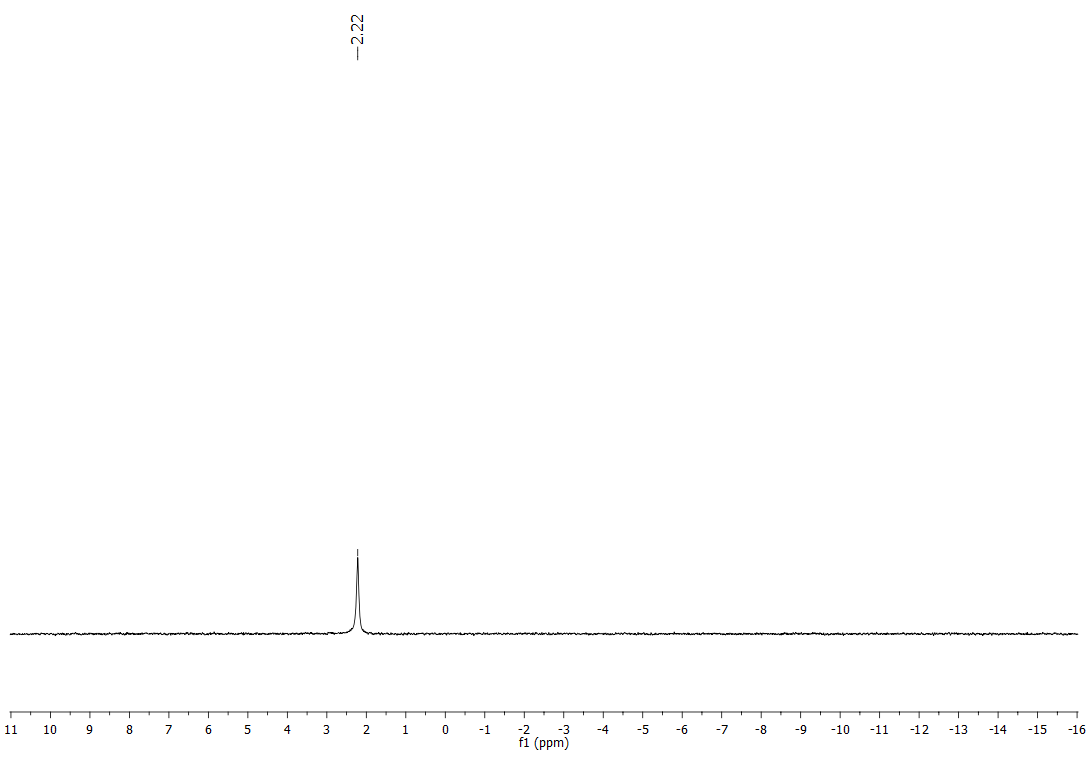


**Figure S23.** ^7^Li-NMR spectrum (155.4MHz, 298K, C_6_D_6_) for complex [{TiCl(^Mes^PDA)}(μ-^Mes^PDA){Li(thf)_2_}] (**4**).


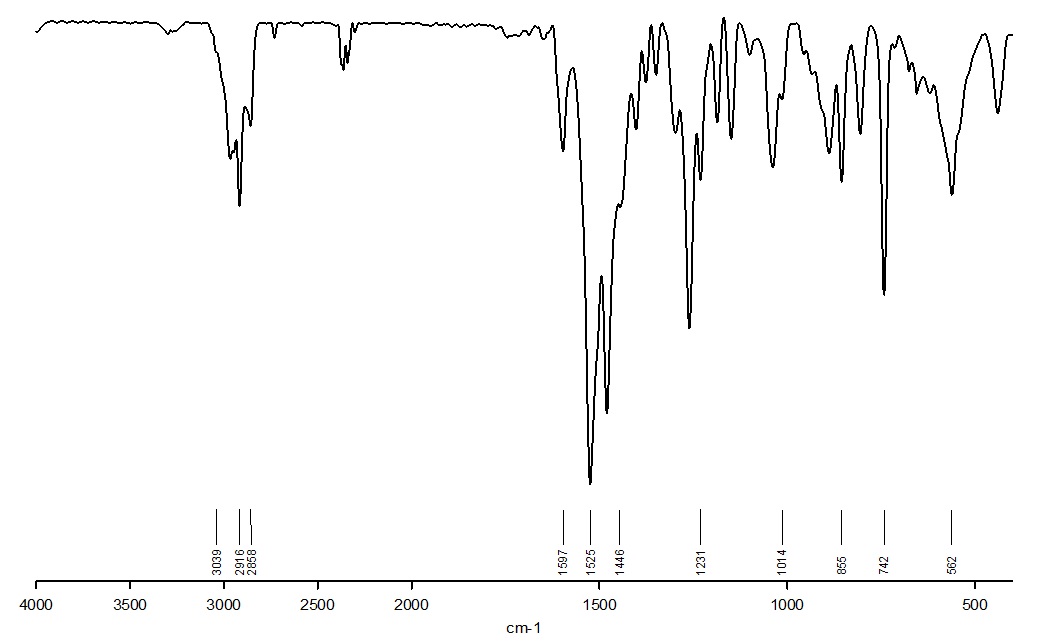


**Figure S24.** FTIR spectrum for complex [{TiCl(^Mes^PDA)}(μ-^Mes^PDA){Li(thf)_2_}] (**4**).


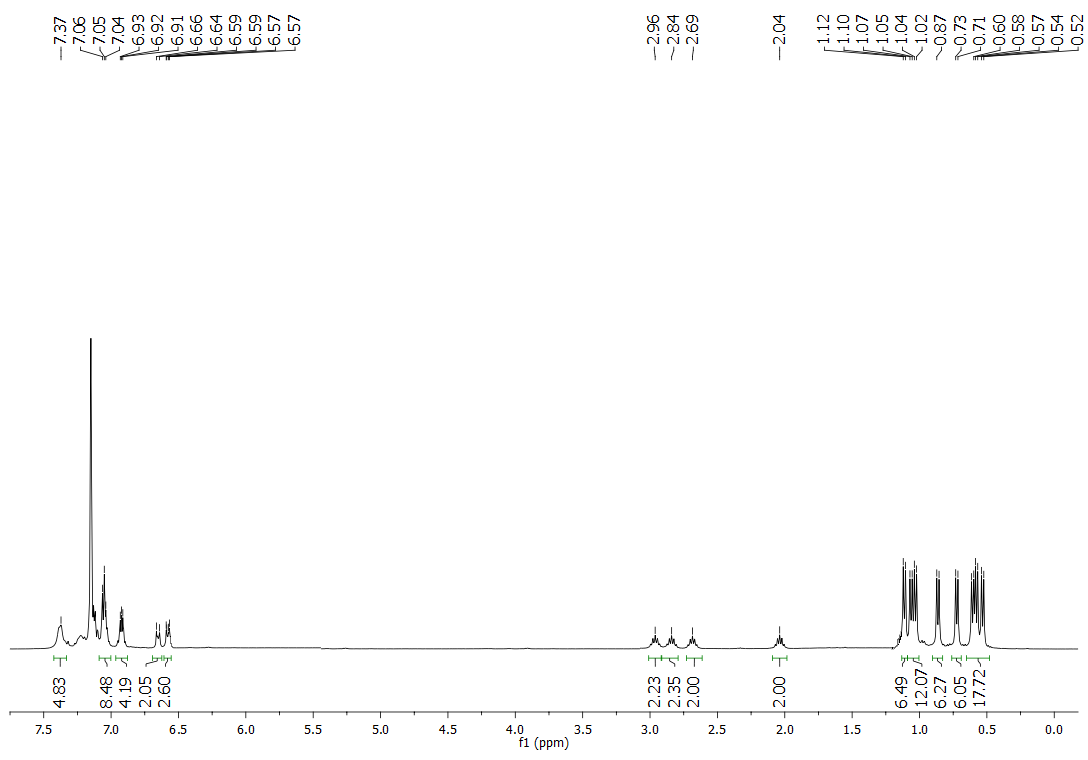


**Figure S25.** ^1^H-NMR spectrum (300MHz, 298K, C_6_D_6_) for complex [Ti(^iPr^PDA)_2_] (**5**).


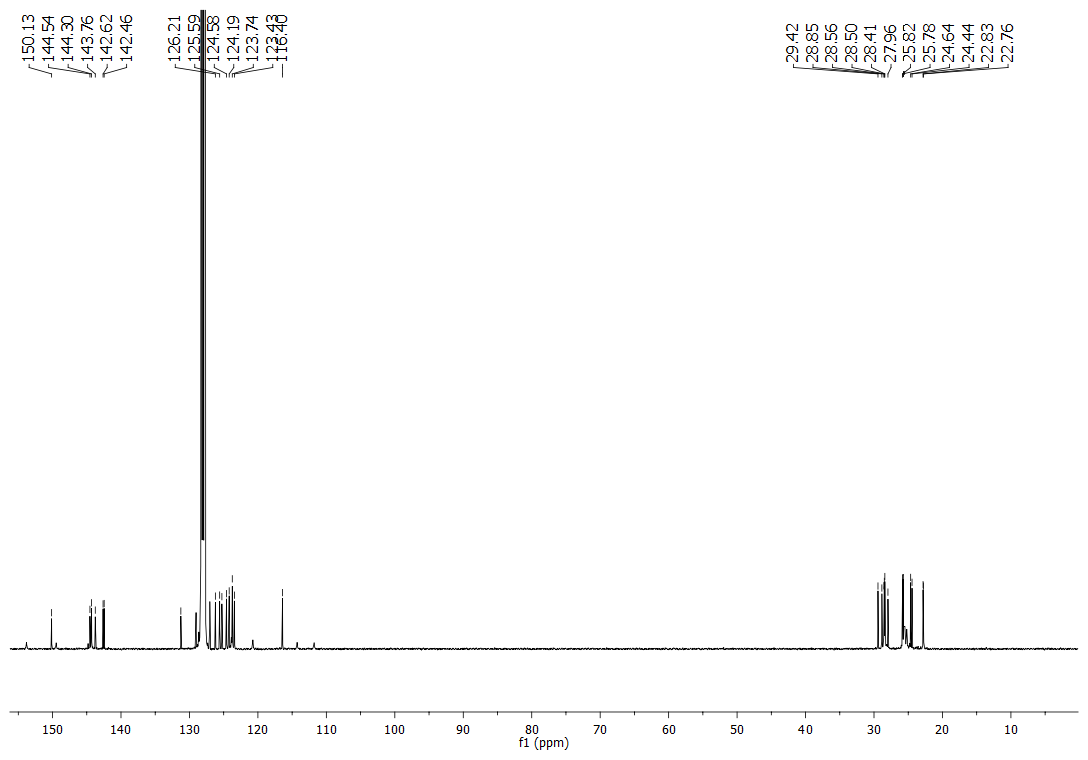


**Figure S26.** ^13^C-{^1^H}-NMR spectrum (75MHz, 298K, C_6_D_6_) for complex [Ti(^iPr^PDA)_2_] (**5**).


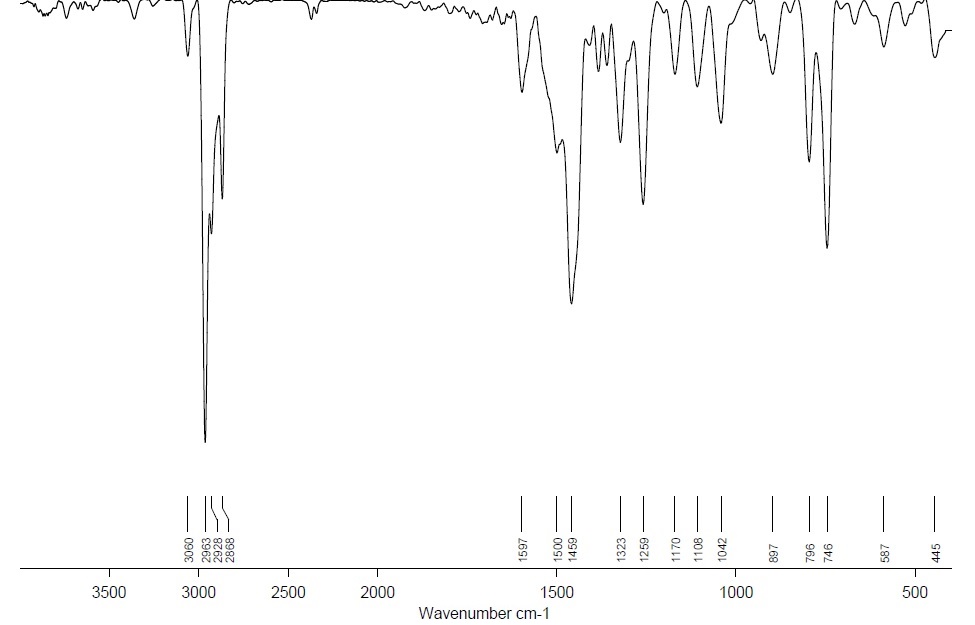


**Figure S27.** FTIR spectrum for complex [Ti(^iPr^PDA)_2_] (**5**).


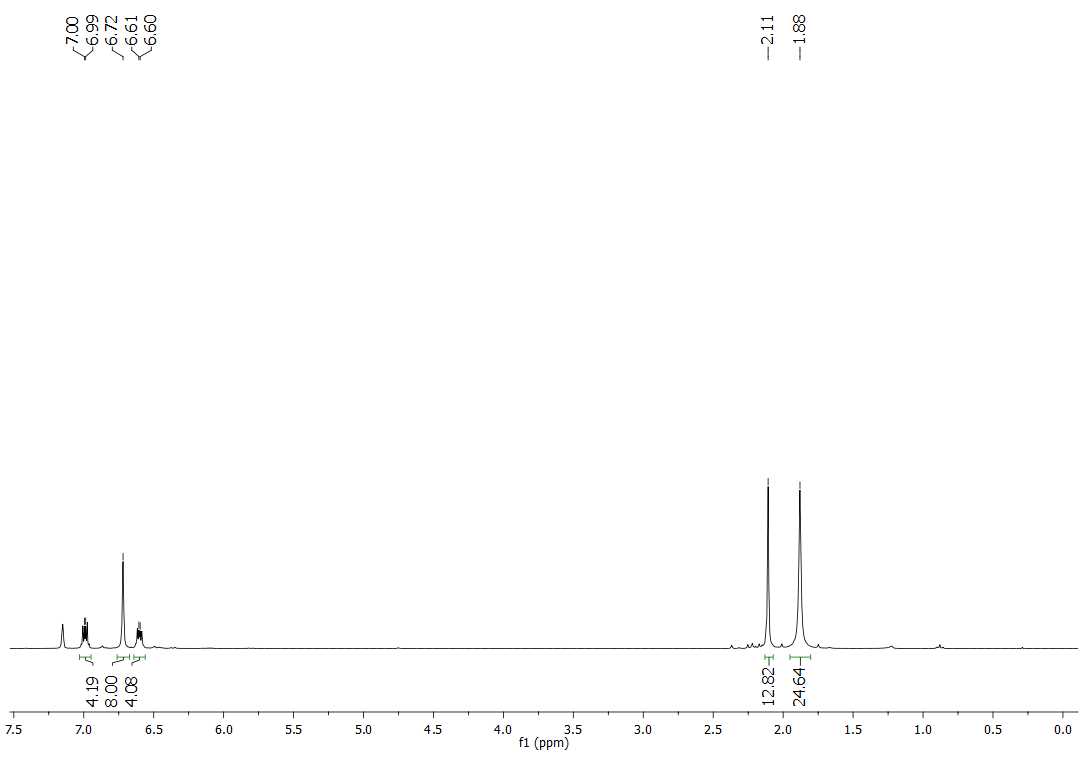


**Figure S28.** ^1^H-NMR spectrum (300MHz, 298K, C_6_D_6_) for complex [Ti(^Mes^PDA)_2_] (**6**).


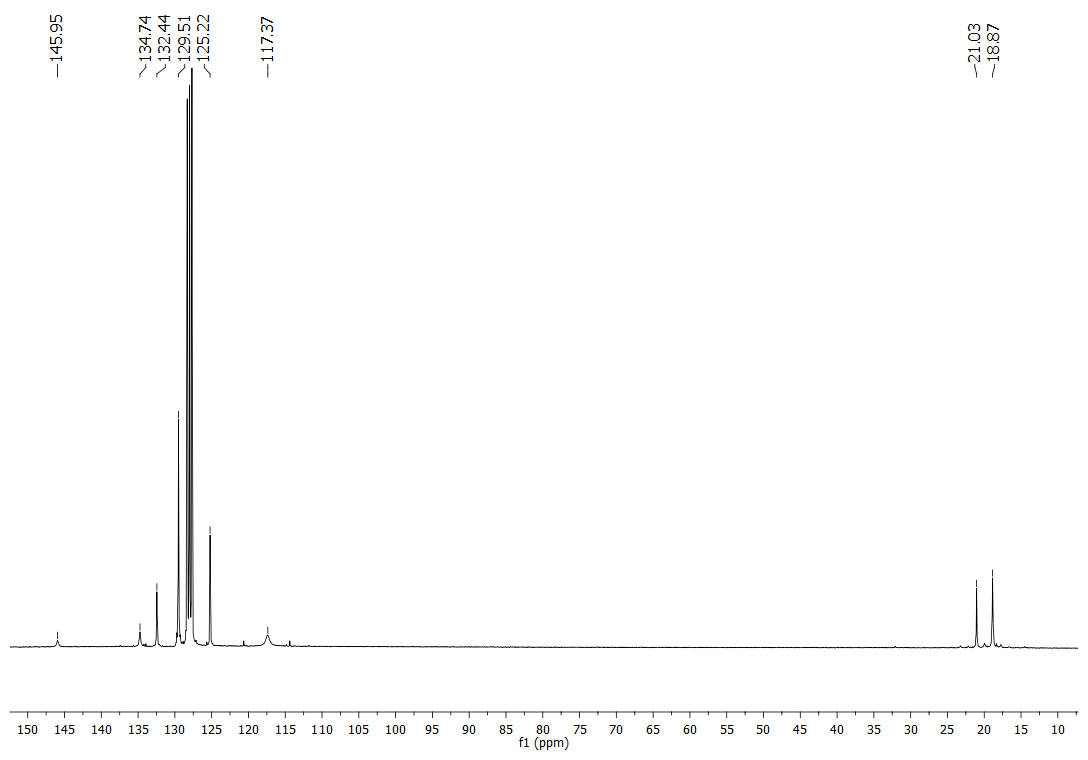


**Figure S29.** ^13^C-{^1^H}-NMR spectrum (75MHz, 298K, C_6_D_6_) for complex [Ti(^Mes^PDA)_2_] (**6**).


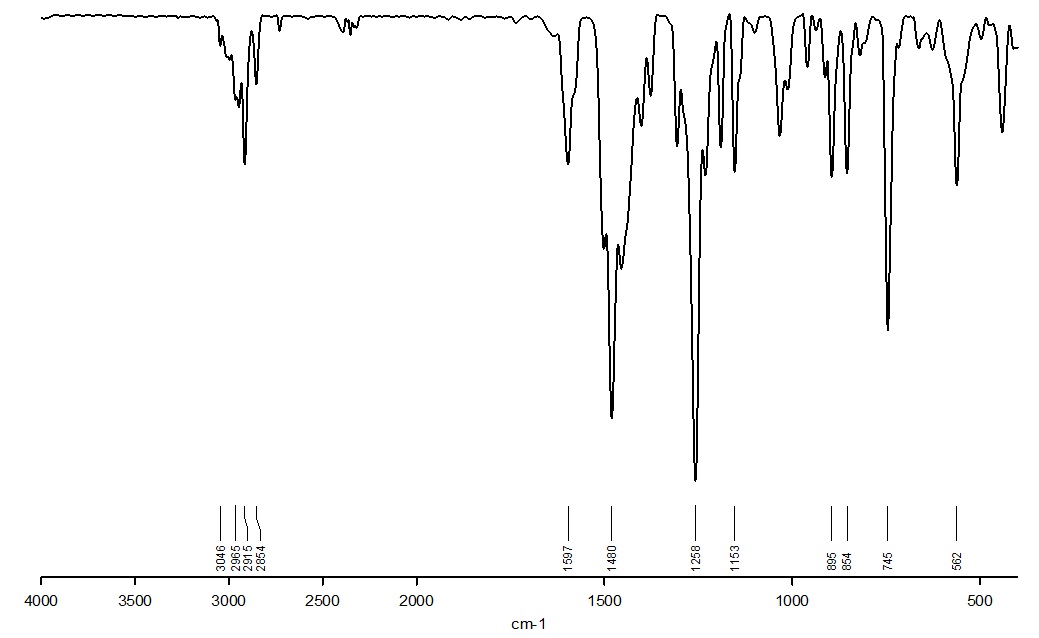


**Figure S30.** FTIR spectrum for complex [Ti(^Mes^PDA)_2_] (**6**)


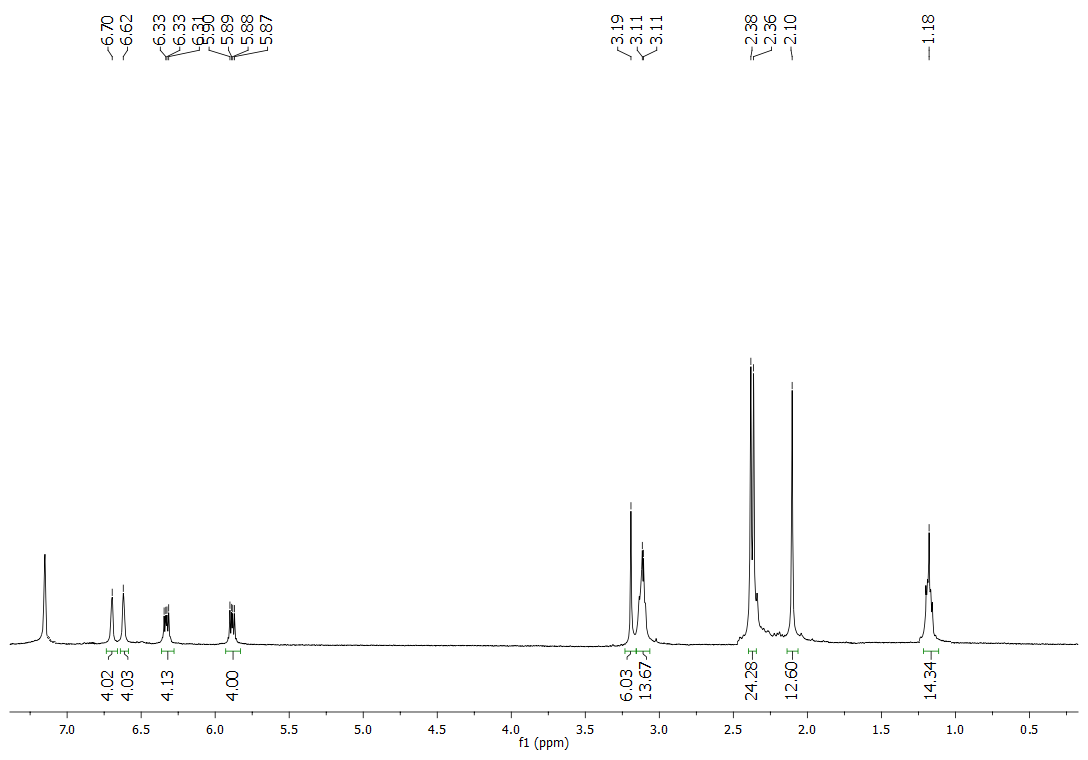


**Figure S31.** ^1^H-NMR spectrum (300MHz, 298K, C_6_D_6_) for complex [Li(thf)_4_][Ti(^Mes^PDA)_2_(N(CH_3_)_2_)] (**7**).


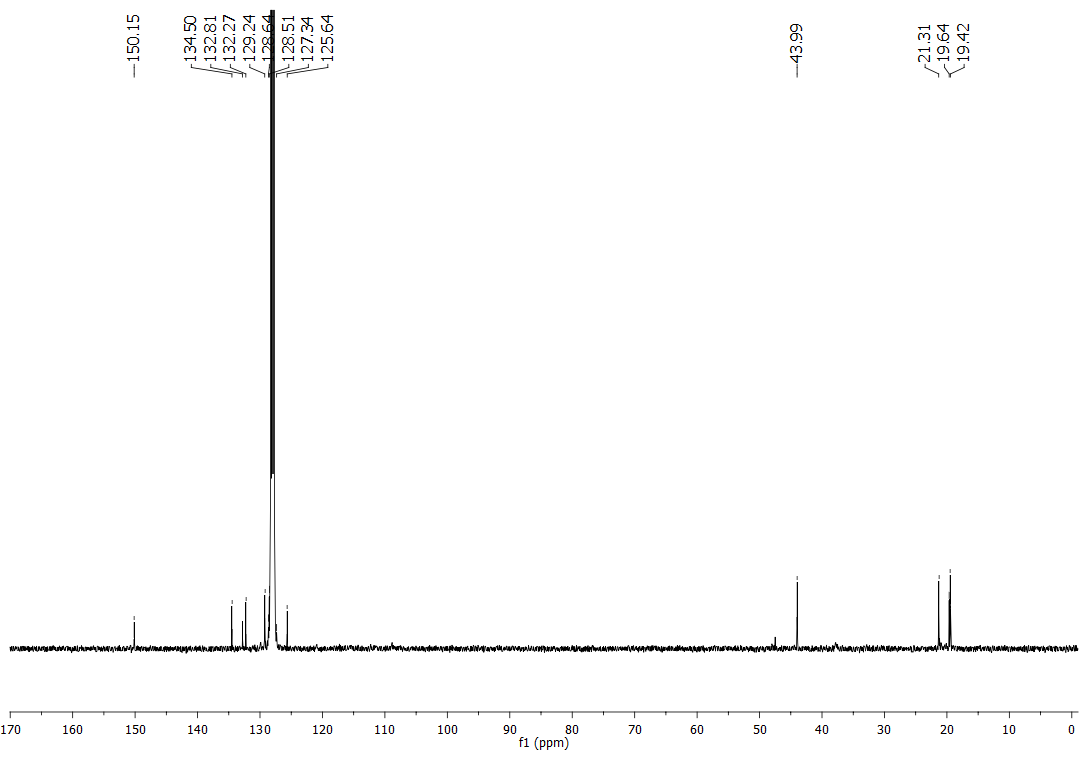


**Figure S32.** ^13^C-{^1^H}-NMR spectrum (75MHz, 298K, C_6_D_6_) for complex [Li(thf)_4_][Ti(^Mes^PDA)_2_(N(CH_3_)_2_)] (**7**).


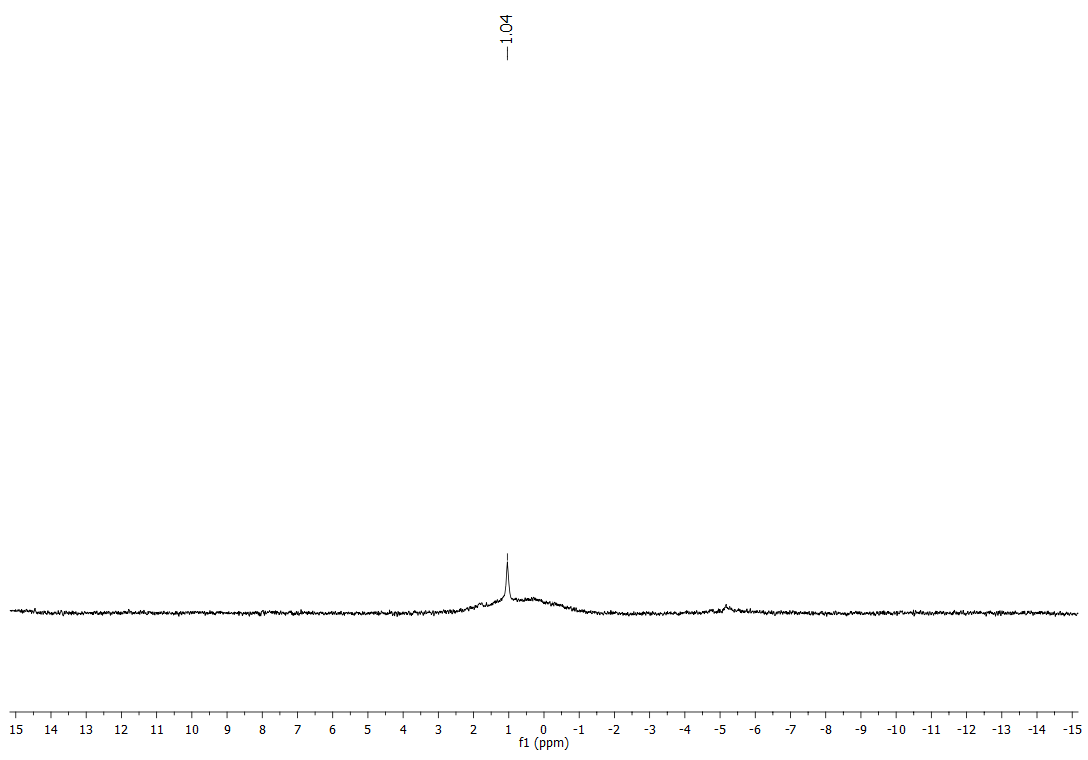


**Figure S33.** ^7^Li-NMR spectrum (155.4 MHz, 298K, C_6_D_6_) for complex [Li(thf)_4_][Ti(^Mes^PDA)_2_(N(CH_3_)_2_)] (**7**).


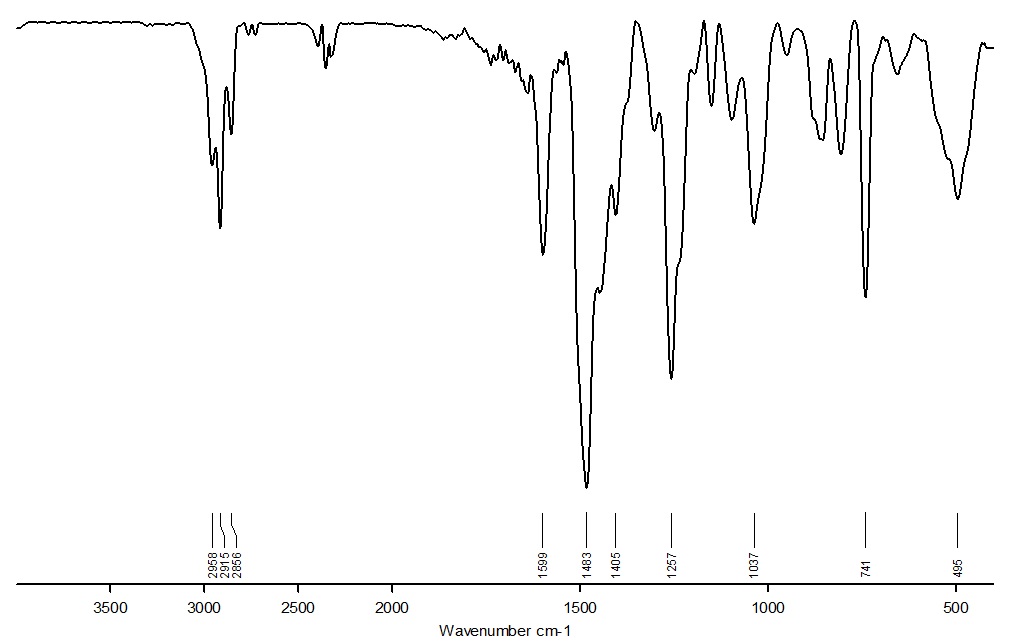


**Figure S34.** FTIR spectrum for complex [Li(thf)_4_][Ti(^Mes^PDA)_2_(N(CH_3_)_2_)] (**7**)


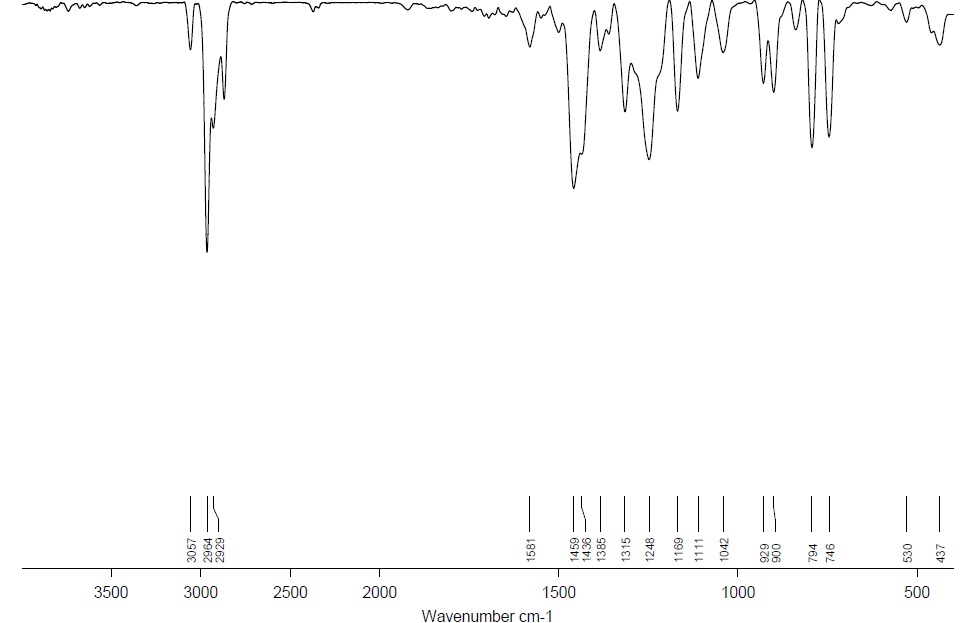


**Figure S35.** FTIR spectrum for complex [Li(thf)_4_][Ti(*^i^*^Pr^PDA)_2_] (**8**)


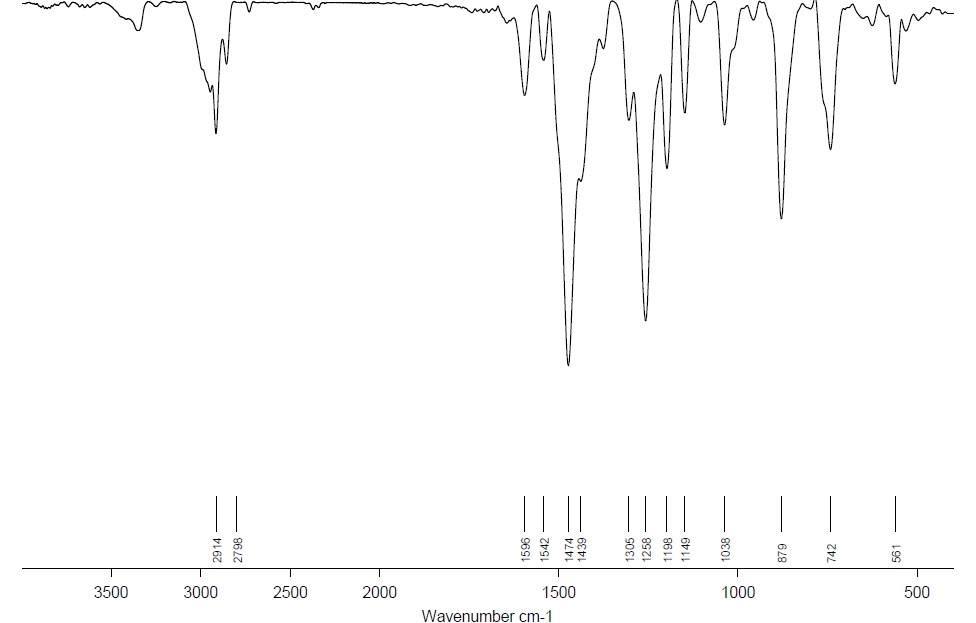


**Figure S36.** FTIR spectrum for complex [Li(thf)_4_][Ti(^Mes^PDA)_2_] (**9**)


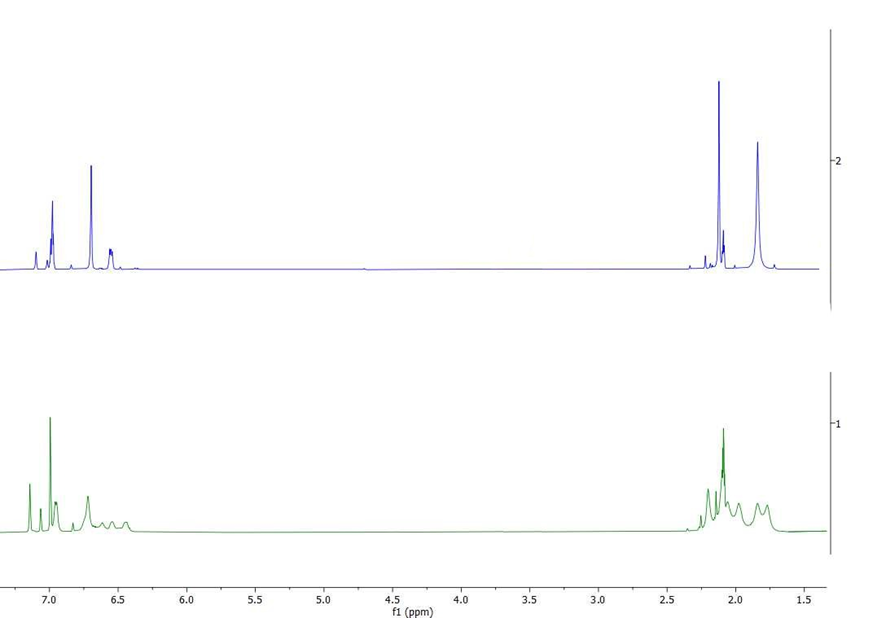


(***a***)

(***b***)

**Figure S37.** Variable temperature ^1^H-NMR spectrum (500MHz, C_7_D_8_) for complex [Ti(^Mes^PDA)_2_] (**6**) at (***a***) 298K, (***b***) 233K

# References

[1] I. Mayer, P. Salvador, Effective atomic orbitals for fuzzy atoms, *J. Chem. Phys.* **2009**, *130*, 234106.

[2] S. Stoll, A. Schweiger, EasySpin, a comprehensive software package for spectral simulation and analysis in EPR, *Journal of Magnetic Resonance* **2006**, *178*, 42-55.

[3] W. J. V. Meerendonk, R. Duchateau, C. E. Koning, G. J. M. Gruter, Unexpected Side Reactions and Chain Transfer for Zinc-Catalyzed Copolymerization of Cyclohexene Oxide and Carbon Dioxide, *Macromolecules,* **2005**, *38*, 7306-7313.
